# Cytotoxicity and genotoxicity of cyphenothrin active ingredient compared with its microencapsulated commercial formulation in human cell lines: an in vitro study

**DOI:** 10.3389/ftox.2026.1932062

**Published:** 2026-10-23

**Authors:** Moustafa Sherif, Abderrahim Nemmar, Bassam R. Ali, Balázs Ádám

**Affiliations:** 1 Institute of Public Health, College of Medicine and Health Sciences, United Arab Emirates University, Al Ain, United Arab Emirates; 2 Department of Anatomy and Embryology, Benha University, Al-Qlayobia, Egypt; 3 Department of Physiology, College of Medicine and Health Sciences, United Arab Emirates University, Al Ain, United Arab Emirates; 4 Department of Genetics and Genomics, College of Medicine and Health Sciences, United Arab Emirates University, Al Ain, United Arab Emirates

**Keywords:** Bombex Farumy, comet assay, cyphenothrin, genotoxicity, HepG2 cells, HL60 cells, microencapsulation, pyrethroid insecticide

## Abstract

**Background:**

Cyphenothrin is a widely used type II pyrethroid insecticide. Its regulatory risk assessment, like that of most pesticides, is based primarily on the active ingredient, leaving the genotoxic potential of encapsulated commercial formulations such as Bombex Farumy largely uncharacterized.

**Methods:**

We compared cytotoxic (propidium iodide exclusion) and genotoxic (alkaline comet assay) effects of cyphenothrin active ingredient *versus* its microencapsulated and released formulations in human HL60 and HepG2 cells following 1 h exposures.

**Results:**

Relative to the active ingredient, DNA damage indicators were numerically lower for encapsulated Bombex Farumy by 29%–83% (median 61%) at concentrations where the active ingredient differed significantly from control. Encapsulated Bombex Farumy showed minimal effects overall, although significant, nonmonotonic increases in tail length and tail moment occurred at select concentrations in HL60, and modest elevations were observed in HepG2 only at 1,000 µM. Released Bombex Farumy showed numerically intermediate effects, but direct pairwise contrasts between released and encapsulated Bombex Farumy were not statistically significant at any concentration or endpoint after correction for multiple comparisons.

**Conclusion:**

In this exploratory, hypothesis generating study, DNA damage indicators were numerically lower for encapsulated than for released formulation or active ingredient cyphenothrin, but direct statistical contrasts between formulations did not reach significance, so a mitigating effect of microencapsulation remains uncertain. Nevertheless, even these preliminary findings suggest that regulatory frameworks relying only on active ingredients may not fully capture formulation specific risks, supporting the need for confirmatory, adequately powered, regulatory compliant testing.

## 1 Introduction

Pesticides have become ubiquitous in modern agriculture and public health applications, yet their widespread use has raised significant concerns regarding potential health and environmental risks (Sherif et al., 2026a; Sherif et al., 2025; Darwish et al., 2026). Despite regulatory efforts, exposure to these chemicals persists across occupational, residential, and environmental contexts, affecting millions worldwide. Pesticide exposure occurs through multiple routes including dermal contact, inhalation, and ingestion, particularly affecting individuals in occupational settings and residents in areas where these chemicals are applied (Sherif et al., 2023).

The regulatory landscape governing pesticides, particularly under frameworks like the US Federal Insecticide, Fungicide, and Rodenticide Act (FIFRA) 2002, prioritizes active ingredients over inert components, despite the latter’s potential biological activity and toxicity (EPA. Pesticides, 2005). This regulatory gap is particularly concerning given that nearly 3,000 substances with varying levels of toxicity are used as inert ingredients in pesticide products in the United States, with approximately 50% considered moderately risky (Cox and Surgan, 2006). However, only a small number of toxicologic tests required for pesticide registration include the complete pesticide formulation, while the majority are conducted with only the active ingredient. This approach fails to assess potential long-term effects of complete formulations, including significant health concerns such as cancer, reproductive problems, and genetic damage (Cox and Surgan, 2006).

Cyphenothrin is a type II pyrethroid insecticide (PubChem. Cyphenothrin National Library of Medicine, 2026) and a cyclopropanecarboxylate ester commonly used for public health vector control and domestic pest management. It demonstrates high efficacy against target pests and is believed to exhibit moderate toxicity to mammals (Lewis et al., 2016). The compound’s mode of action involves modulation of sodium channels through its cyano group, leading to nerve membrane depolarization and subsequent pest mortality (PubChem. Cyphenothrin National Library of Medicine, 2026). The lowest no observed adverse effect level (NOAEL) derived from studies on cyphenothrin was determined to be 3 mg/kg body weight per day, with the Food Safety Commission of Japan (FSCJ) specifying an acceptable daily intake (ADI) of 0.015 mg/kg body weight per day after applying a safety factor of 200 (FSC-Japan, 2023). Cyphenothrin was specifically selected for this study due to the demonstrated genotoxic potential of related pyrethroids. Studies have shown that pyrethroids such as phenothrin, cypermethrin, permethrin, and deltamethrin exhibit DNA damaging effects in various cellular systems (Amjad et al., 2018; Villarini et al., 1998; Mahmoud et al., 2026). These studies have generally relied on short term *in vitro* exposures, typically ranging from 30 min to a few hours, at concentrations spanning the low to high micromolar range; for example, our group’s parallel work on permethrin found the pure active ingredient cytotoxic at 2,300 μM, while its microencapsulated formulation showed no measurable cytotoxicity across the same concentration range yet still induced significant genotoxicity. However, comprehensive genotoxicity data for cyphenothrin itself, especially comparative assessment of the active ingredient with its product formulations, remain limited despite its widespread use in domestic and public health applications.

Recent advancements in pesticide technology have introduced novel delivery systems, including micro- and nano-encapsulation, designed to optimize efficacy while potentially reducing acute exposure risks (Ramadhan Makame et al., 2023). Encapsulated formulations, like Bombex Farumy in case of cyphenothrin, offer theoretical advantages including controlled release and reduced immediate toxicity. However, these technological innovations present new challenges, as their altered toxicological profiles and potential for chronic exposure effects necessitate thorough investigation (Ramadhan Makame et al., 2023). The assumption that encapsulation universally reduces toxicity requires empirical validation through systematic genotoxicity assessments.

Genetic damage assessment has emerged as a critical component of pesticide safety evaluation, as chromosomal aberrations represent important events in mutation development and carcinogenesis (Martus et al., 2015).

The toxicological evaluation of pesticides, specifically regarding their genotoxic and carcinogenic properties, represents a crucial aspect in developing appropriate preventive measures (Villaverde et al., 2018). The increasing prevalence of novel commercial pesticides and encapsulated formulations with limited safety information underscores the need for enhanced scrutiny. As per the European Environmental Agency (EEA) report, many chemicals are available in the market, but only a minor proportion of these chemicals have undergone comprehensive evaluation for their potential risks (EEA, 2020).

Given the widespread detection of pesticides in environmental samples as they are found in all streams, more than 70% of common foods, and in over half of adults and children in monitoring programs (Cox and Surgan, 2006), understanding the comparative genotoxicity of active ingredients *versus* commercial formulations becomes critical. Children aged 3–6 years represent a particularly vulnerable population due to increased dermal contact and non-dietary oral ingestion during play activities (Jurewicz et al., 2015), emphasizing the urgency of comprehensive safety assessments, especially in case of pesticides that may be domestically used such as cyphenothrin.

Despite cyphenothrin’s widespread domestic and agricultural use, no previous studies have comparatively assessed the genotoxic potential of its active ingredient *versus* its microencapsulated commercial formulation in human cell systems. While related pyrethroids have demonstrated DNA damaging effects (Amjad et al., 2018; Villarini et al., 1998), cyphenothrin specific genotoxicity data, particularly comparing active ingredients with product formulations, remain absent from the scientific literature. This knowledge gap is particularly concerning given that regulatory frameworks predominantly evaluate active ingredients in isolation, potentially misrepresenting actual human health risks from commercial products. This study addresses this critical knowledge gaps by systematically evaluating the cytotoxic and genotoxic effects of cyphenothrin active ingredient compared to its commercial micro-encapsulated formulation, Bombex Farumy, in human white blood cells and liver cells. By employing comet assay, we aim to determine whether encapsulation technology truly mitigates genotoxic potential, by identifying the minimum concentrations required to cause significant DNA damage. Our study is the first to provide this critical comparative assessment using human cell lines, directly addressing this regulatory blind spot and providing empirical evidence for formulation specific toxicological evaluation.

These findings will inform preliminary evidence that can inform evidence based regulatory decisions and contribute to safer pesticide development practices, ultimately protecting human public health while maintaining agricultural productivity. Our results should be viewed as hypothesis generating data that highlight the need for comprehensive formulation specific testing using validated regulatory protocols, including standardized battery tests required by OECD guidelines (OECD, 2023; OECD, 2016). The comet assay, while not a stand-alone regulatory endpoint, serves as a valuable indicator test that complements standard genotoxicity batteries by detecting early DNA damage events that may not be captured by regulatory assays such as bacterial reverse mutation or chromosomal aberration tests (Kirkland and Speit, 2008; Burlinson et al., 2007). These preliminary findings justify and necessitate follow-up investigations using regulatory compliant methodologies to confirm formulation specific genotoxicity patterns.

## 2 Materials and methods

### 2.1 Test chemicals

Cyphenothrin, obtained as a 25 mg package (Cod.Art.: CRM1Y6P138) with a lot number of 221LACH181, demonstrated an assay purity of 98.3% with a tolerance uncertainty of 0.5%. Its Chemical Abstracts Service (CAS) number is 39151-40-7, with a molecular formula of C_24_H_25_NO_3_ and a molecular weight of 375.46 g/mol. This compound was sourced from Lab Instruments S. r.l., High Pure Standard and Chemical Division. To prepare a stock concentration of 10 mM (equivalent to 10,000 μM), 25 mg of cyphenothrin powder was dissolved in 6.6585 mL of methanol. From this stock solution, serial dilutions in RPMI cell culture medium were prepared for the cytotoxicity assay to final cyphenothrin concentrations of 0.1, 1, 10, 100, 200, 300, 500, 750 and 1,000 μM (HL60) or 0.1, 1, 10, 100, 200, 500, 1,000, 2000 and 4,000 μM (HepG2); the genotoxicity assay used the subset of these concentrations meeting the ≥70% viability criterion: 0.1, 1, 10, 50, 100, 150 and 200 μM in HL60 cells, and 0.01, 0.1, 1, 10, 100, 500 and 1,000 μM in HepG2 cells.

Bombex Farumy, a product manufactured by Jesmond, contains cyphenothrin at a concentration of 10.70% and prallethrin at 1.10%, with the remaining 88.20% comprising other ingredients. According to the product label, it contains cyphenothrin at a concentration of 100 g/L, microencapsulated based on advanced co-microencapsulation (CS) technology, corresponding to 0.266 mol/L considering its molecular weight of 375.46 g/mol. Prallethrin is present at 10 g/L as a free active ingredient.

To test Bombex Farumy in its encapsulated form, 37.5 μl of the product formulation was mixed with 962 μl of RPMI complete medium to achieve a concentration of 10,000 μM. Variable concentrations of encapsulated Bombex Farumy ranging from 0.01 to 1,000 μM were derived by diluting the stock solution into RPMI complete medium.

*To prepare a working stock concentration of 10 mM of released Bombex Farumy,* 1.54 ml *of the original formulation of Bombex Farumy was mixed with* 2.55 ml *of methanol, yielding an intermediate mixture of approximately 100 mM based on the product’s labelled cyphenothrin content*. The protocol for preparation involves incubation of the mix for 2 h with continuous vortexing, then centrifuging for another 2 h at 2500Xg at room temperature (RT). After centrifugation and removal of solids, the resulting liquid layer was diluted 10 fold to yield the final 10 mM stock solution used for subsequent serial dilutions. The 10 mM methanol stock was used solely for accurate gravimetric dissolution of cyphenothrin, and it was not applied directly to cells at this concentration. Each working solution was prepared directly to its final target concentration in a total volume of 1 mL, which was applied to the well without further dilution, so the nominal test concentration equals the actual in well concentration. Because the stock was 10,000 μM in 100% methanol, this means the methanol fraction prior to the 30 min evaporation step was C/100% at a nominal concentration of C μM, 10% at the highest active ingredient concentration tested (1,000 μM), 1% at 100 μM, and negligible (<0.01%) at ≤10 μM. All working concentrations were prepared by serial dilution into RPMI complete medium. Following preparation, all methanol based working solutions (including the cyphenothrin active ingredient serial dilutions and the released Bombex Farumy dilutions) were kept open in a laminar flow hood for 30 min under sterile conditions to allow passive evaporation of residual methanol prior to cell exposure. Matched vehicle controls, prepared and evaporated identically, were included at all concentration points for both test formulations and showed no significant cytotoxic or genotoxic effects compared to untreated PBS controls (data not shown). This confirms that observed effects are attributable to the test compounds. From this stock solution, escalating concentrations of released Bombex Farumy (0.01, 0.1, 1, 10, 100, 200, 500, and 1,000 μM) were prepared by diluting the stock solution within RPMI complete medium.

### 2.2 Cell cultures

Two human cell lines were employed as *in vitro* models representing the primary target tissues of pesticide genotoxicity. The HL 60 cell line was kindly provided by Dr. Mahmood Al Mashhadani (College of Medicine, Mohammed Bin Rashid University of Medicine and Health Sciences, Dubai, UAE) and is commercially derived from AddexBio (San Diego, CA, USA; Catalogue #C0003023). The HepG2 cell line was obtained *via* collaboration with Professor Bassam R. Ali (a co-author) at the College of Medicine and Health Sciences, United Arab Emirates University, who originally acquired them from the American Type Culture Collection (ATCC, Manassas, VA, USA; Catalogue # HB-8065). Both collaborating laboratories sourced the cells directly from their respective commercial repositories and provided them to our team under the terms of their Material Transfer Agreements. As this study did not involve primary human samples or participants, institutional human subjects ethics approval was not required.

These cell lines were selected as the most widely used *in vitro* models for leukocytes and hepatocytes, representing the two most important target cell types for pesticide toxicity evaluation in addition to tissues at portals of entry. Both cell lines were maintained in RPMI 1640 medium (Cat#: 11875093, Gibco) supplemented with 10% fetal bovine serum (Cat#: A5256701, Gibco) and 1% Penicillin-Streptomycin (10,000 U/mL) (Cat#: 15140122, Gibco). The HL60 cells were cultured as floating cells in suspension, while HepG2 cells were grown as an adherent monolayer. All cultures received fresh medium 24 h before experimentation to maintain optimal cell conditions.

### 2.3 Cell treatment

Cells were exposed to escalating doses of cyphenothrin, Bombex Farumy in both encapsulated and released forms in the cell culture medium. Genotoxicity assay concentrations were selected from those that maintained cell viability at ≥70% in the cytotoxicity assessment. This approach is consistent with concentration selection principles used in other *in vitro* OECD genotoxicity guidelines (OECD, 2023). It helps ensure that the measured DNA damage reflects genotoxic effects, not a marked cytotoxicity. This selection process is depicted schematically in Figure 1. Various concentrations of the two substances, along with PBS as a negative control, were introduced into the cell cultures and maintained for 1 h at 37 °C. The concentration of PBS in the cell culture medium consistently remained below 10% (v/v). Hydrogen peroxide at a concentration of 100 μM served as the positive control. Cells were kept at a low density and provided with fresh medium 24 h before genotoxicity analysis.

**FIGURE 1:**
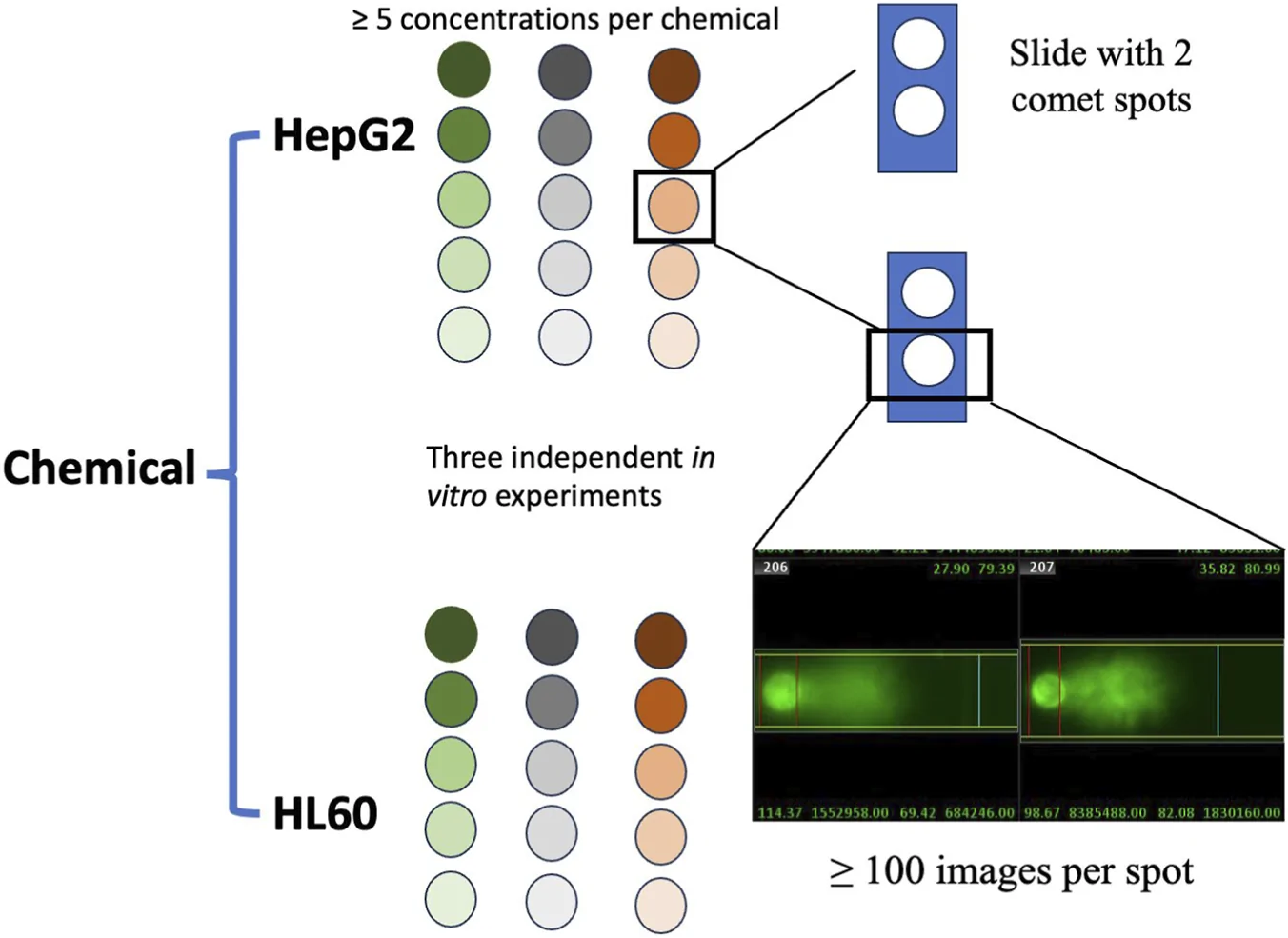
Experimental design and data analysis workflow. Schematic representation of the experimental workflow for cytotoxicity and genotoxicity assessment. HL60 and HepG2 cells were treated with increasing concentrations of the test chemicals. Based on cytotoxicity results, selected concentrations were used for the comet assay to assess genotoxicity. Each slide contained two spots per experiment, and each sample was measured in duplicate, one positioned at the front and one at the back of the electrophoresis apparatus. For comet analysis, only slides with more than 100 cells per concentration per experiment were included. Adapted from Sherif et al. (2026) with permission (Sherif et al., 2026b).

### 2.4 Cytotoxicity assay

Following treatment, samples were promptly assessed for cytotoxicity as previously described (Mahmoud et al., 2026; Sherif et al., 2026b). Propidium iodide (PI) fluorescent dye was employed for cell labeling. PI, a DNA intercalating dye, permeates membranes of deceased and dying cells while being unable to penetrate the plasma membranes of viable, healthy cells.

The PI fluorescent dye was dissolved in PBS (pH 7.2) to achieve a final concentration of 2 μM. Subsequently, 200 μL of this working solution was added to cell pellets containing 1 × 10^5 cells and incubated for 30 min at 4 °C, shielded from light. Following labeling, the cells were washed and re-suspended in ice cold PBS buffer. Then, 40 μL of the cell suspension was placed on a microscope slide for immediate examination under a Zeiss epifluorescent microscope at ×20 magnification.

To ascertain the total cell count, transmitted light microscopy was utilized on two non overlapping fields per slide to determine the survival rate through visual inspection. Concurrently, the TRITC filter for PI was applied to excite the labeled cells. Cell viability was calculated as a percentage using the formula: Viability (%) = [(total cell count − PI positive cell count)/total cell count] × 100. The mean of the percentages of living cells from repeated experiments represented cell viability.

### 2.5 Genotoxicity assay

The alkaline version of the comet assay, based on the methodology outlined by Collins (2004), was performed (Collins, 2004). Post treatment, samples underwent centrifugation, and HL60 cells were resuspended in PBS at a density of 1800cells/μL. Because HepG2 cells grow adherently, they were first briefly trypsinized (approximately 1 min) with shaking of the flask to detach the monolayer, after which trypsin activity was stopped by addition of 10x complete RPMI medium, and the resulting cell suspension was then processed identically to HL60 cells from the centrifugation step onward and resuspended in PBS at the same density. Frosted degreased slides were coated with a single layer comprising 1% normal melting point agarose (NMA) overlaid with 0.75% low melting point agarose (LMA) containing the cells (2 × 10^5 per slide). A coverslip 20 × 20 mm was applied on the top to allow proper spreading of the LMA cells mix over the slide. Following 10 min solidification at 4 °C, the coverslips were removed, and embedded cells underwent a prolonged lysis process using CometAssay Lysis Solution (Cat#4250-050-01) at 4 °C for overnight, shielded from light. Subsequently, the DNA was permitted to unwind for 20 min in alkaline unwinding solution (300 mM NaOH, 1 mM EDTA) and then subjected to electrophoresis in the same solution for 20 min at 1 V/cm and 300 mA. Finally, the slides were gently rinsed twice with Milli Q water to eliminate residual alkali and detergent and washed with 70% ethanol two times. After drying for 10 min in dark, each slide was stained with PI (20 μg/ml), washed with water for 1 minute, covered with coverslip and stored in a humidified container until analysis.

Fluorescence signals were captured at ×40 magnification using the Metafer automated image analysis system from MetaSystems GmbH, Germany, equipped with ZEISS Axio Imager Z2 and cometscan module. The Neon Software (MetaSystems GmbH, Germany) facilitated the analysis of >100 randomly captured comet images from duplicate slides per each concentration and computation of DNA damage parameters. Consistent with comet assay guidance (OECD. OECD Organisation for Economic Co-operation and Development, 2016; Azqueta and Collins, 2013; Lorenzo et al., 2013), heavily damaged ‘hedgehog/ghost’ comets were identified visually and excluded from the quantitative % tail DNA analysis, and were scored separately because % tail DNA is unreliable for these extreme profiles. This exclusion was applied during the Neon Software analysis pipeline. Tail DNA%, tail length in μm, tail moment, and olive moment were quantified to assess DNA damage. Percentage of DNA in the tail measures the DNA quantity in the tail, tail length indicates how far DNA has migrated from the nucleus, tail moment is a combination of tail length and the proportion of DNA in the tail, and Olive tail moment accounts for tail length and the distance between the centers of mass of the head and tail. Results were presented as the mean of the median values of DNA damage parameters from at least three independent experiments, with statistical analysis conducted on these experiment level medians, not on individual cell level values.

### 2.6 Data analysis

The experiments were performed independently three times (Figure 1), each using the same cell lines but different passages. Cytotoxicity was calculated as one minus the cell viability, represented by the mean proportion of living cells from these independent experiments.

For comet analysis, only slides containing more than 100 images per concentration for each experiment were considered, with slides containing fewer images (typically due to processing artifacts) discarded from the analysis, and exclusion events were not individually logged. The median of comet assay metrics (DNA percentage in the tail, tail length, tail moment, and Olive moment) at various concentrations of the test formulations were calculated by Microsoft Excel 2021. The arithmetic mean of the medians from the independent experiments, statistical analyses, and generation of figures and tables were performed using RStudio (version 2025.09.2 + 418, Cucumberleaf Sunflower; Posit PBC, Boston, MA, USA) with the following packages: readxl (v1.4.3) for data import, ggplot2 (v3.5.1) for figure generation, and multcomp (v1.4-26) for Dunnett’s *post hoc* contrasts; all packages were sourced from CRAN (https://cran.r-project.org).

Because comet assay data at the individual cell level are well documented to be strongly right skewed, the per-experiment median was used as the unit submitted to parametric testing. This two step median then mean of medians approach is consistent with established comet assay statistical practice (OECD. OECD (Organisation for Economic Co-operation and Development), 2016; Bright et al., 2011; Lovell et al., 1999) and is the primary basis for the parametric ANOVA/Dunnett approach used throughout. As a supplementary check, normality (Shapiro Wilk) and variance homogeneity (Levene’s) diagnostics were run on the resulting experiment level values for every chemical x concentration x cell line x endpoint combination; 11% of comet assay combinations only failed one or both assumptions. Given this small sample instability, and for consistency and comparability across combinations, ANOVA with Dunnett’s *post hoc* test was retained uniformly throughout.

The arithmetic means of cell viability and of the median values of comet assay metrics from repeated experiments at various concentrations were compared to those of untreated cells using ANOVA followed by Dunnett’s *post hoc* test. For direct pairwise comparisons between the released and encapsulated Bombex Farumy formulations at each concentration, additional contrasts were performed within the same ANOVA framework. To control the family wise error rate across these multiple pairwise comparisons, p values were adjusted using the Holm sequential Bonferroni method. Statistical significance for this formulation *versus* formulation contrasts was determined using Holm adjusted p values. A statistically significant difference was considered at a 5% significance level. The error bars in the figures represent ±1 standard error (SE) to illustrate the variability of the data across different experiments.

IC50 values (concentration reducing viability to 50% of control) were estimated using nonlinear regression with a four parameter logistic model (where data permitted). For formulations where viability did not decline below 50% within the tested concentration range, IC50 was reported as exceeding the maximum tested concentration.

## 3 Results

### 3.1 Cytotoxicity assessments

The cytotoxicity assessment following 1 h exposure demonstrated concentration dependent responses for all test chemicals in HL60 cells (Figure 2A). Cell viability was maintained above 80% at concentrations up to 100 μM, indicating minimal acute cytotoxicity within this range. However, significant differences in cytotoxic potency emerged at higher concentrations.

**FIGURE 2:**
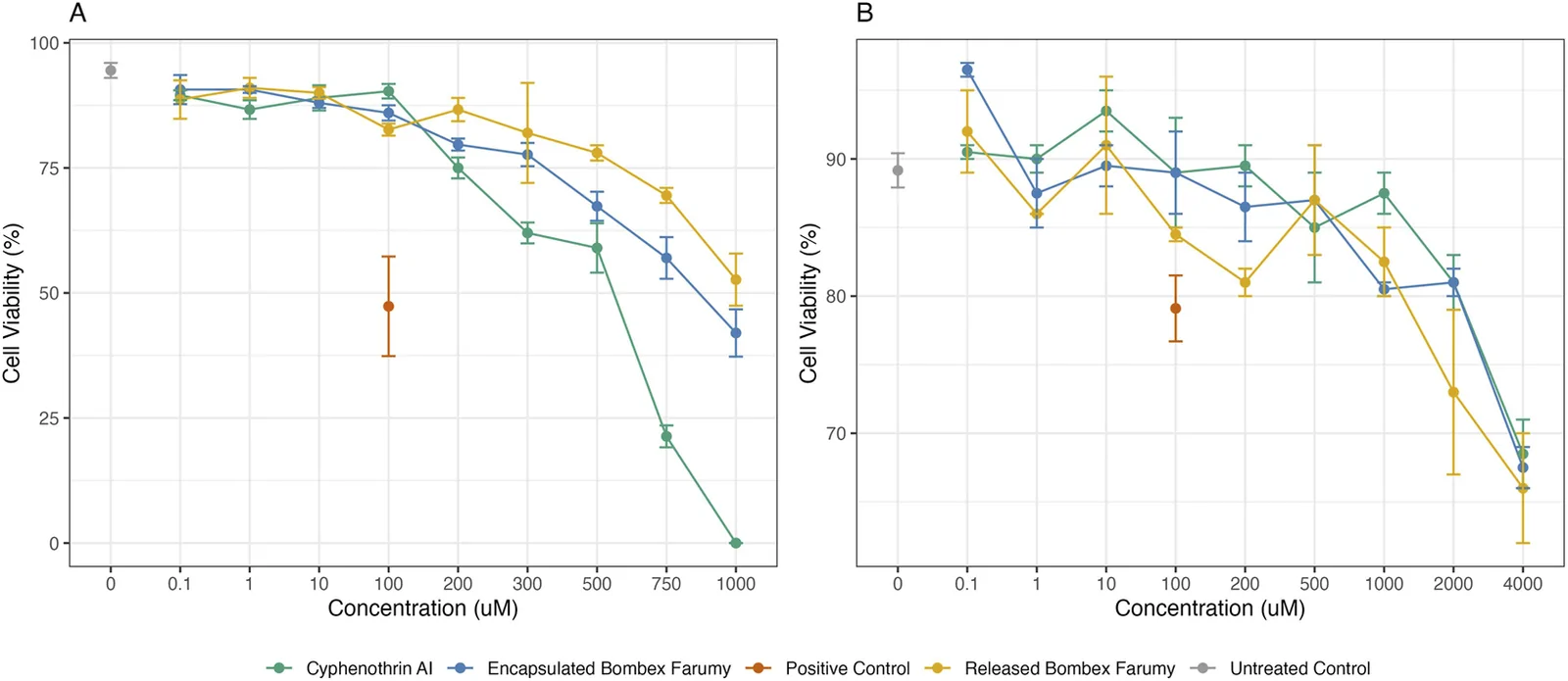
Cytotoxic effects of cyphenothrin and its formulation on HL60 **(A)** and HepG2 **(B)** cells. The graph shows cell viability (%) following 1‐h exposure to various concentrations (HL60: 0.1–1,000 M; HepG2: 0.1–4,000 M) of cyphenothrin active ingredient and its encapsulated formulation Bombex Farumy in original and released form. Error bars represent standard error of the mean of three independent experiments (one-way ANOVA with Dunnett’s *post hoc* test vs. untreated control). Asterisks indicate statistically significant differences compared to untreated control (*p < 0.05, **p < 0.01, ***p < 0.001); exact values are presented in Supplementary Table S1.

Released Bombex Farumy showed numerically higher mean viability than encapsulated Bombex Farumy at every concentration from 200 μM up to the highest tested concentration (mean difference from control at 1,000 μM: −41.83 for released vs. −52.50 for encapsulated; Supplementary Table S1); however, a direct statistical contrast between the two formulations at each concentration did not reach significance at any point after correction for multiple comparisons (Holm-adjusted p ≥ 0.42 throughout). Encapsulated Bombex Farumy showed an intermediate cytotoxicity profile, remaining above 70% viability up to 300 μM before declining further at higher concentrations. In stark contrast, cyphenothrin active ingredient displayed the most pronounced cytotoxicity, with a sharp significant decline in viability from 200 μM, reaching complete cell death (0% viability) at 1,000 μM.

Statistical analysis revealed the first significant reduction in cell viability at 200 μM for both cyphenothrin active ingredient (mean difference [MD]: 19.50 ± 3.681, p < 0.001) and encapsulated Bombex Farumy (MD: 14.83 ± 4.145, p = 0.013), while released Bombex Farumy showed significant cytotoxicity only at 500 μM (MD: 16.50 ± 5.056, p = 0.028); viability continued to decline significantly at every higher concentration tested for all three test formulations (Table 1). IC50 (the concentration reducing viability to 50% of control) could be reliably estimated only for cyphenothrin active ingredient (792.29 μM, 95% CI 473.77–1,110.82 μM), the only test chemical whose viability declined through the 50% threshold within the concentrations tested; for encapsulated Bombex Farumy, mean viability fell just below 50% at the highest tested concentration (1,000 μM: 42.0%), and for released Bombex Farumy it remained just above (52.7%). In both cases the four-parameter logistic model did not converge to a stable, within-range estimate, so IC50 for both formulations is reported as exceeding 1,000 μM.

**TABLE 1:** Statistically significant cytotoxicity and genotoxicity effects of cyphenothrin active ingredient, encapsulated Bombex Farumy, and released Bombex Farumy relative to untreated control, by cell line, endpoint, and concentration.

Assay	Cell line	Endpoint	Chemical	Conc. (µM)	Mean diff. ± SE	p (vs. control)	p (4 endpoint Holm)
Cytotoxicity	HL60	% viability	Cyphenothrin AI	200	−19.50 ± 3.681	<0.001	n/a
Cytotoxicity	HL60	% viability	Cyphenothrin AI	300	−32.50 ± 3.681	<0.001	n/a
Cytotoxicity	HL60	% viability	Cyphenothrin AI	500	−35.50 ± 3.681	<0.001	n/a
Cytotoxicity	HL60	% viability	Cyphenothrin AI	750	−73.17 ± 3.681	<0.001	n/a
Cytotoxicity	HL60	% viability	Cyphenothrin AI	1,000	−94.50 ± 3.681	<0.001	n/a
Cytotoxicity	HL60	% viability	Released Bombex Farumy	500	−16.50 ± 5.056	0.028	n/a
Cytotoxicity	HL60	% viability	Released Bombex Farumy	750	−25.00 ± 5.539	0.002	n/a
Cytotoxicity	HL60	% viability	Released Bombex Farumy	1,000	−41.83 ± 5.056	<0.001	n/a
Cytotoxicity	HL60	% viability	Encapsulated Bombex Farumy	200	−14.83 ± 4.145	0.013	n/a
Cytotoxicity	HL60	% viability	Encapsulated Bombex Farumy	300	−16.83 ± 4.145	0.004	n/a
Cytotoxicity	HL60	% viability	Encapsulated Bombex Farumy	500	−27.17 ± 4.145	<0.001	n/a
Cytotoxicity	HL60	% viability	Encapsulated Bombex Farumy	750	−37.50 ± 4.145	<0.001	n/a
Cytotoxicity	HL60	% viability	Encapsulated Bombex Farumy	1,000	−52.50 ± 4.145	<0.001	n/a
Cytotoxicity	HepG2	% viability	Cyphenothrin AI	2000	−8.17 ± 2.559	0.050	n/a
Cytotoxicity	HepG2	% viability	Cyphenothrin AI	4,000	−20.67 ± 2.559	<0.001	n/a
Cytotoxicity	HepG2	% viability	Released Bombex Farumy	2000	−16.17 ± 3.437	0.003	n/a
Cytotoxicity	HepG2	% viability	Released Bombex Farumy	4,000	−23.17 ± 3.437	<0.001	n/a
Cytotoxicity	HepG2	% viability	Encapsulated Bombex Farumy	1,000	−8.67 ± 2.439	0.025	n/a
Cytotoxicity	HepG2	% viability	Encapsulated Bombex Farumy	2000	−8.17 ± 2.439	0.037	n/a
Cytotoxicity	HepG2	% viability	Encapsulated Bombex Farumy	4,000	−21.67 ± 2.439	<0.001	n/a
Genotoxicity	HL60	% DNA in tail	Cyphenothrin AI	100	26.17 ± 6.805	0.013	0.025
Genotoxicity	HL60	% DNA in tail	Cyphenothrin AI	200	27.67 ± 6.086	0.004	0.005
Genotoxicity	HL60	Tail length (µm)	Cyphenothrin AI	100	57.00 ± 9.753	<0.001	0.002
Genotoxicity	HL60	Tail length (µm)	Cyphenothrin AI	150	62.00 ± 9.753	<0.001	<0.001
Genotoxicity	HL60	Tail length (µm)	Cyphenothrin AI	200	51.33 ± 8.723	<0.001	0.001
Genotoxicity	HL60	Tail length (µm)	Released Bombex Farumy	50	34.00 ± 6.700	0.002	0.010
Genotoxicity	HL60	Tail length (µm)	Released Bombex Farumy	100	46.00 ± 6.700	<0.001	0.001
Genotoxicity	HL60	Tail length (µm)	Released Bombex Farumy	200	28.00 ± 6.700	0.010	0.039
Genotoxicity	HL60	Tail length (µm)	Encapsulated Bombex Farumy	1	33.50 ± 6.486	0.001	0.005
Genotoxicity	HL60	Tail length (µm)	Encapsulated Bombex Farumy	50	28.00 ± 6.486	0.005	0.021
*Genotoxicity*	*HL60*	*Tail length (µm)*	*Encapsulated Bombex Farumy*	*100*	*21.33 ± 5.802*	*0.017*	0.069*
Genotoxicity	HL60	Tail length (µm)	Encapsulated Bombex Farumy	150	31.00 ± 6.486	0.002	0.009
Genotoxicity	HL60	Tail moment	Cyphenothrin AI	100	30.83 ± 5.808	<0.001	0.003
Genotoxicity	HL60	Tail moment	Cyphenothrin AI	150	27.83 ± 5.808	0.002	0.007
Genotoxicity	HL60	Tail moment	Cyphenothrin AI	200	30.67 ± 5.195	<0.001	0.001
*Genotoxicity*	*HL60*	*Tail moment*	*Released Bombex Farumy*	*100*	*20.83 ± 6.089*	*0.034*	0.101*
*Genotoxicity*	*HL60*	*Tail moment*	*Encapsulated Bombex Farumy*	*1*	*10.83 ± 3.495*	*0.049*	0.147*
*Genotoxicity*	*HL60*	*Tail moment*	*Encapsulated Bombex Farumy*	*150*	*11.33 ± 3.495*	*0.038*	0.113*
Genotoxicity	HL60	Olive tail moment	Cyphenothrin AI	100	8.17 ± 2.576	0.043	0.043
*Genotoxicity*	*HL60*	*Olive tail moment*	*Cyphenothrin AI*	*150*	*8.17 ± 2.576*	*0.043*	0.086*
Genotoxicity	HL60	Olive tail moment	Cyphenothrin AI	200	11.00 ± 2.304	0.002	0.005
Genotoxicity	HepG2	% DNA in tail	Cyphenothrin AI	1,000	28.39 ± 5.330	<0.001	<0.001
Genotoxicity	HepG2	% DNA in tail	Released Bombex Farumy	1,000	35.26 ± 6.980	<0.001	<0.001
*Genotoxicity*	*HepG2*	*Tail length (µm)*	*Cyphenothrin AI*	*100*	*23.54 ± 8.056*	*0.045*	0.181*
*Genotoxicity*	*HepG2*	*Tail length (µm)*	*Cyphenothrin AI*	*500*	*26.22 ± 8.056*	*0.020*	0.078*
Genotoxicity	HepG2	Tail length (µm)	Cyphenothrin AI	1,000	38.00 ± 8.056	<0.001	<0.001
Genotoxicity	HepG2	Tail length (µm)	Released Bombex Farumy	1,000	30.11 ± 8.143	0.007	0.007
Genotoxicity	HepG2	Tail moment	Cyphenothrin AI	1,000	29.63 ± 5.102	<0.001	<0.001
Genotoxicity	HepG2	Tail moment	Released Bombex Farumy	1,000	31.68 ± 7.048	<0.001	0.002
*Genotoxicity*	*HepG2*	*Tail moment*	*Encapsulated Bombex Farumy*	*1,000*	*16.64 ± 5.584*	*0.039*	0.118*
Genotoxicity	HepG2	Olive tail moment	Cyphenothrin AI	1,000	10.44 ± 1.760	<0.001	<0.001
Genotoxicity	HepG2	Olive tail moment	Released Bombex Farumy	1,000	15.62 ± 2.743	<0.001	<0.001
*Genotoxicity*	*HepG2*	*Olive tail moment*	*Encapsulated Bombex Farumy*	*1,000*	*5.85 ± 1.767*	*0.017*	0.067*
Cytotoxicity	HL60	% viability	Positive control (100 μM H2O2)	100	−47.20 ± 10.043	0.039	n/a
*Cytotoxicity*	*HepG2*	*% Viability*	*Positive Control (100 μM H2O2)*	*100*	*−10.10 ± 2.709*	*0.091*	*n/a*
Genotoxicity	HL60	% DNA in tail	Positive control (100 μM H2O2)	100	14.00 ± 4.819	0.048	0.048
Genotoxicity	HL60	Tail length (µm)	Positive control (100 μM H2O2)	100	32.30 ± 4.596	0.015	0.046
Genotoxicity	HL60	Tail moment	Positive control (100 μM H2O2)	100	12.03 ± 1.702	0.004	0.017
Genotoxicity	HL60	Olive tail moment	Positive control (100 μM H2O2)	100	4.34 ± 1.105	0.020	0.046
Genotoxicity	HepG2	% DNA in tail	Positive control (100 μM H2O2)	100	45.70 ± 3.492	0.005	0.014
Genotoxicity	HepG2	Tail length (µm)	Positive control (100 μM H2O2)	100	57.40 ± 5.083	<0.001	0.002
*Genotoxicity*	*HepG2*	*Tail moment*	*Positive Control (100 μM H2O2)*	*100*	*57.92 ± 5.566*	*0.038*	0.064*
*Genotoxicity*	*HepG2*	*Olive tail moment*	*Positive Control (100 μM H2O2)*	*100*	*22.19 ± 1.793*	*0.032*	0.064*

IC50 (HL60): cyphenothrin AI 792.29 μM (95% CI 473.77–1,110.82 μM); encapsulated and released Bombex Farumy exceed 1,000 μM (model fits unstable beyond the tested range). IC50 (HepG2): all three test formulations exceed 4,000 μM. Direct Holm-corrected released vs. encapsulated contrasts reached significance at none of the 74 concentration × endpoint × cell line combinations tested (smallest Holm-adjusted p = 0.42, HL60 cytotoxicity at 500 μM). Entries marked with an asterisk (*) did not remain significant after a further Holm correction applied across the four comet endpoints tested per chemical × cell line × concentration. Positive control (100 μM H2O2) results are included for reference; because the positive control was tested at a single concentration and was not part of the Dunnett multiple comparison family used for the three test chemicals, its p values were instead obtained from a two sample Welch’s t-test against untreated control, with the same four endpoint Holm correction applied to its genotoxicity p values for consistency.

The cytotoxicity assessment in HepG2 cells after 1 h exposure revealed markedly different concentration response relationships compared to HL60 cells, with overall reduced sensitivity to cyphenothrin induced cell death (Figure 2B). All three chemicals maintained cell viability above 80% up to 500 μM, with significant responses emerging only at higher concentrations.

Both cyphenothrin active ingredient and encapsulated Bombex Farumy demonstrated remarkably stable cytotoxicity profiles, maintaining cell viability above 80% up to 2000 μM and declining below 70% only at 4,000 μM. Released Bombex Farumy exhibited somewhat more pronounced cytotoxic effects, with viability declining more steeply to below 80% at 2000 μM and reaching approximately 66% at 4,000 μM.

Statistical significance in cell viability reduction was first observed at 1,000 μM and 2000 μM for encapsulated and released Bombex Farumy (p < 0.05), respectively, while the active ingredient also induced significant cell death at 2000 μM (p = 0.050), with further pronounced effects at 4,000 μM (p < 0.001). Despite these statistical differences, the overall patterns of the dose response curves remained similar across test chemicals. For the same reason, IC50 could not be reliably estimated for any of the three test formulations in HepG2 cells, since viability remained above 50% throughout the entire tested concentration range (0.1–4,000 μM); IC50 for all three therefore exceeds 4,000 μM.

### 3.2 Genotoxicity assessments

The comet assay revealed different genotoxic profiles of the test chemicals across the four measured parameters, with cyphenothrin active ingredient consistently inducing the most severe DNA damage (Figure 3).

**FIGURE 3:**
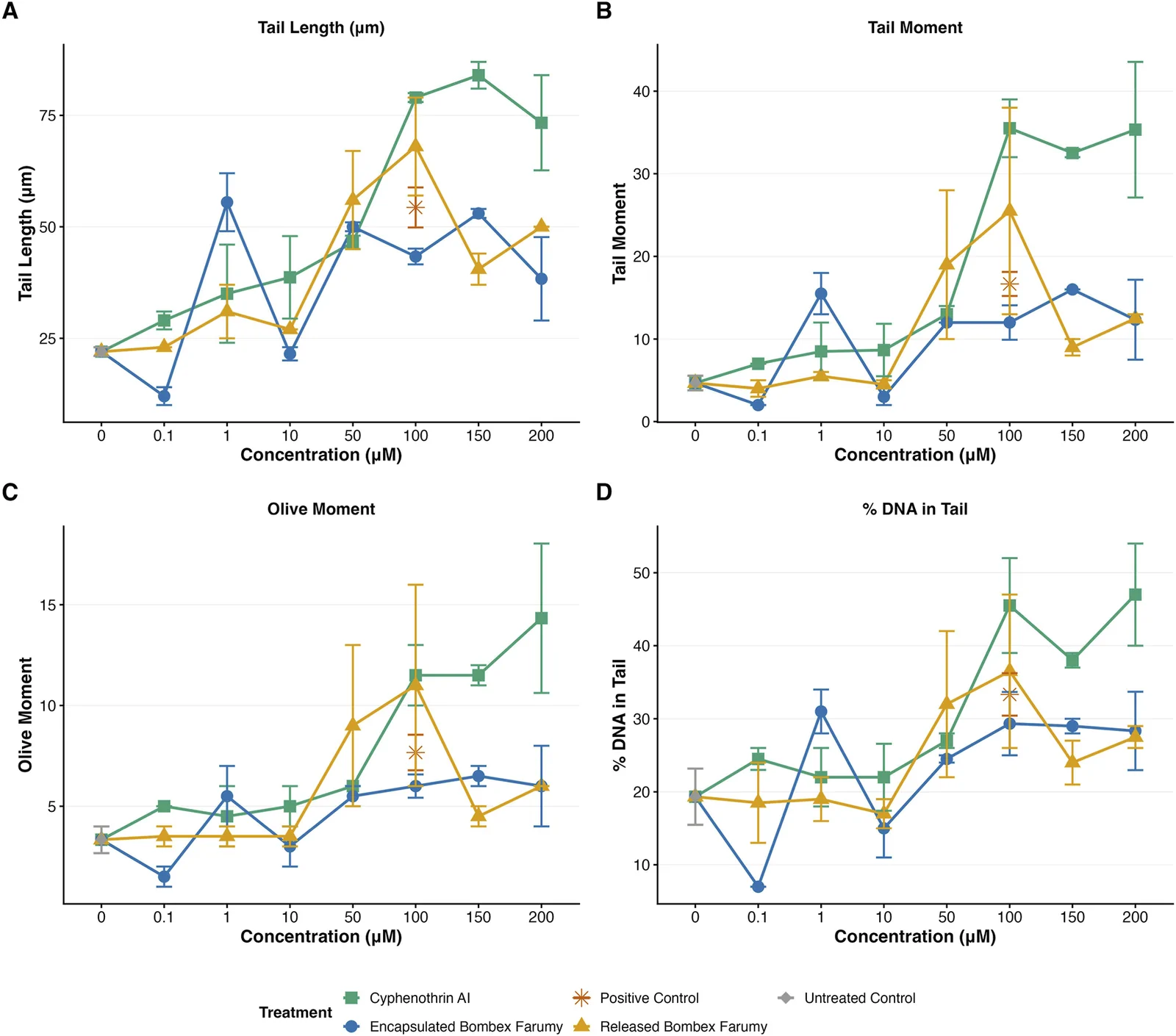
Genotoxic effects of cyphenothrin and its formulation on HL60 cells. The graphs show four comet assay parameters: **(A)** tail length, **(B)** tail moment, **(C)** Olive tail moment, and **(D)** % DNA in tail measured after 1-h exposure to different concentrations (0–200 µM) of cyphenothrin active ingredient and its encapsulated and released formulation Bombex Farumy. Error bars represent standard error of the mean of three independent experiments (one-way ANOVA with Dunnett’s *post hoc* test vs. untreated control). Asterisks indicate statistically significant differences compared to untreated control (*p < 0.05, **p < 0.01, ***p < 0.001); exact values are presented in Supplementary Table S1.

Cyphenothrin active ingredient induced the highest levels of DNA migration, with statistically significant increases in DNA percentage in the tail at 100 μM (MD: 26.17 ± 6.805, p = 0.013) and 200 μM (MD: 27.67 ± 6.086, p = 0.004) compared to untreated controls. Remarkably, neither the encapsulated nor released forms of Bombex Farumy demonstrated significant increases in DNA percentage in tail at any tested concentration.

Cyphenothrin active ingredient also exhibited concentration dependent increases in tail length, with significant effects at 100 μM (MD: 57.00 ± 9.753 μm, p < 0.001), 150 μM (MD: 62.00 ± 9.753 μm, p < 0.001), and 200 μM (MD: 51.33 ± 8.723 μm, p < 0.001). Released Bombex Farumy demonstrated significant increases at 50 μM (MD: 34.00 ± 6.700 μm, p = 0.002), 100 μM (MD: 46.00 ± 6.700 μm, p < 0.001), and 200 μM (MD: 28.00 ± 6.700 μm, p = 0.010). Encapsulated Bombex Farumy showed significant increases in tail length at 1.0 μM (MD: 33.50 ± 6.486, p = 0.001), 50.0 μM (MD: 28.00 ± 6.486, p = 0.005), 100.0 μM (MD: 21.33 ± 5.802, p = 0.017), and 150.0 μM (MD: 31.00 ± 6.486, p = 0.002), indicating a consistent, though non monotonic, concentration dependent response pattern.

For tail moment, cyphenothrin active ingredient induced significant increases at 100 μM (MD: 30.83 ± 5.808, p < 0.001), 150 μM (MD: 27.83 ± 5.808, p = 0.002), and 200 μM (MD: 30.67 ± 5.195, p < 0.001). Released Bombex Farumy showed significant DNA damage only at 100 μM (MD: 20.83 ± 6.089, p = 0.034), while encapsulated Bombex Farumy showed significant increases in tail moment at 1.0 μM (MD: 10.83 ± 3.495, p = 0.049) and 150.0 μM (MD: 11.33 ± 3.495, p = 0.038), though not at intermediate concentrations.

Olive tail moment was also significantly increased by cyphenothrin active ingredient at 100 μM (MD: 8.17 ± 2.576, p = 0.043), 150 μM (MD: 8.17 ± 2.576, p = 0.043), and 200 μM (MD: 11.00 ± 2.304, p = 0.003). Neither the released nor the encapsulated Bombex Farumy formulations demonstrated significant effects in Olive tail moment in the tested concentration range.

Collectively, the active ingredient consistently induced the most pronounced DNA damage across all parameters, followed by released Bombex Farumy, while encapsulated Bombex Farumy exhibited the least genotoxicity.

The genotoxicity evaluation in HepG2 cells revealed concentration thresholds substantially higher than those observed in HL60 cells, with significant effects generally appearing at 1,000 μM across multiple parameters (Figure 4).

**FIGURE 4:**
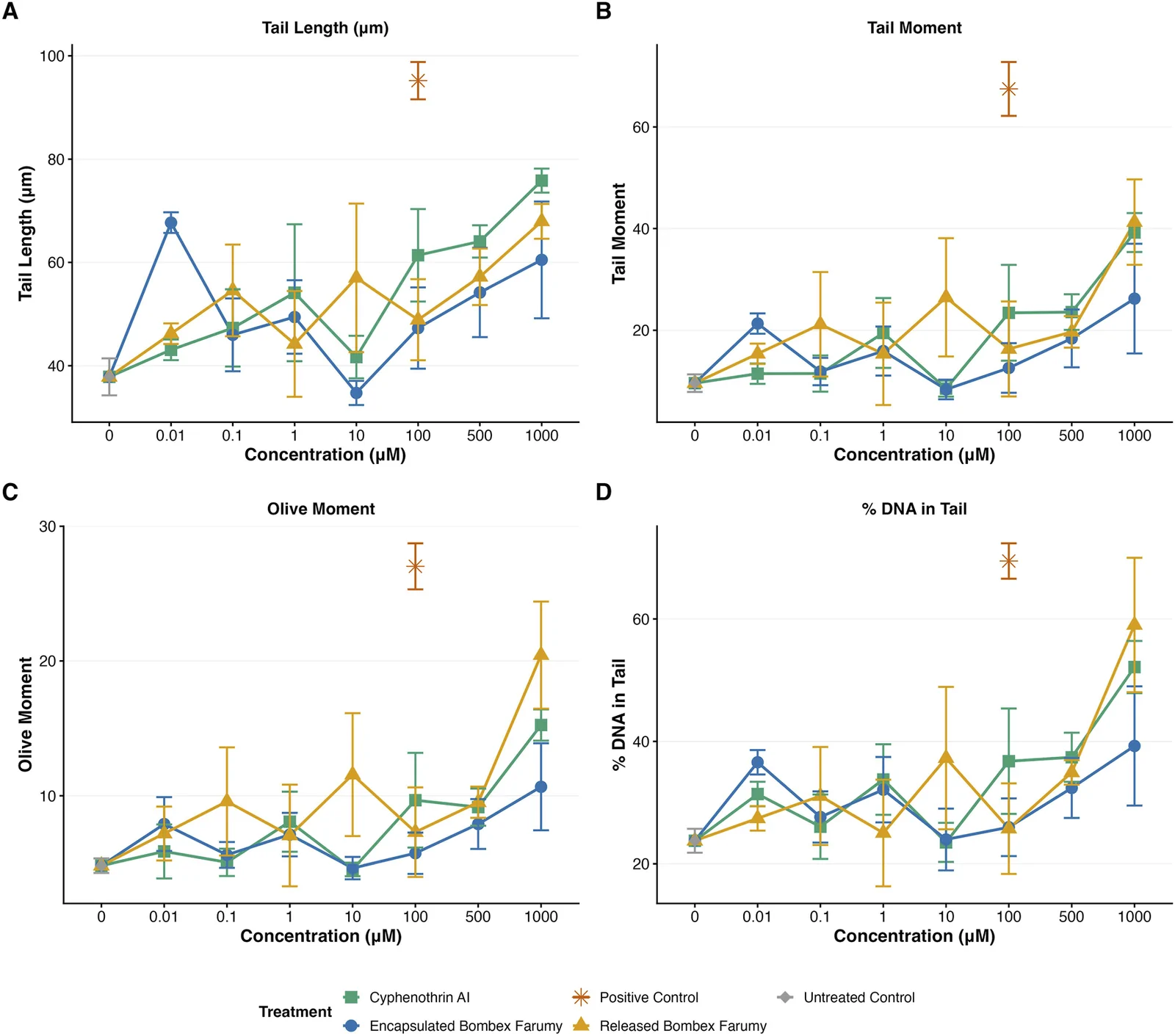
Genotoxic effects of cyphenothrin formulations on HepG2 cells. The graphs show four comet assay parameters: (A) tail length, (B) tail moment, (C) Olive tail moment, and (D) % DNA in tail measured after 1-h exposure to different concentrations (0–1,000 µM) of cyphenothrin active ingredient and Bombex Farumy in encapsulated and released form. Error bars represent standard error of the mean of three independent experiments (one-way ANOVA with Dunnett’s *post hoc* test vs. untreated control). Asterisks indicate statistically significant differences compared to untreated control (*p < 0.05, **p < 0.01, ***p < 0.001); exact values are presented in Supplementary Table S1.

Cyphenothrin active ingredient induced significant increases of DNA percentage in tail at 1,000 μM (MD: 28.39%, p < 0.001), similar to released Bombex Farumy (MD: 35.26%, p < 0.001). Encapsulated Bombex Farumy failed to demonstrate statistically significant increases at any tested concentration.

Significant increases in tail length were observed for cyphenothrin active ingredient starting at 100 μM (MD: 23.54 ± 8.056, p = 0.045), continuing at 500 μM (MD: 26.22 ± 8.056, p = 0.020), and most robustly at 1,000 μM (MD: 38.00 ± 8.056, p < 0.001). Released Bombex Farumy also demonstrated a significant increase in tail length at 1,000 μM (MD: 30.11 μm, p = 0.007), while encapsulated Bombex Farumy did not reach statistical significance at any tested concentration (p = 0.092 at 1,000 μM).

Tail moment was significantly increased by cyphenothrin active ingredient at 1,000 μM (MD: 29.63, p < 0.001). Released Bombex Farumy also demonstrated significant elevation at the same concentration (MD: 31.68, p = 0.001), and encapsulated Bombex Farumy similarly showed a significant increase at 1,000 μM (MD: 16.64, p = 0.039), though of lesser magnitude than both the active ingredient and the released formulation.

For Olive tail moment, the results confirmed the established patterns, with cyphenothrin active ingredient exhibiting significant increases at 1,000 μM (MD: 10.44, p < 0.001). Released Bombex Farumy also showed significant elevation at 1,000 μM (MD: 15.62, p < 0.001), while encapsulated Bombex Farumy demonstrated the lowest values across all concentrations, though a significant increase was observed at the highest tested concentration of 1,000 μM (MD: 5.85, p = 0.017).

Table 1 summarizes every statistically significant treatment effect (one way ANOVA with Dunnett’s *post hoc* test vs. untreated control) observed across both cell lines and both assays, organized by chemical, cell line, endpoint, and concentration, together with the 100 μM H2O2 positive control results for each endpoint. For genotoxicity, an additional Holm correction was applied across the four comet endpoints (tail length, tail moment, Olive tail moment, %DNA in tail) tested per chemical × cell line × concentration; entries marked with an asterisk (*) and shown in italics do not remain significant after this correction. Full concentration response data, including non significant concentrations, are provided in Supplementary Table S1.

## 4 Discussion

This study provides the first comparative assessment of cyphenothrin and its microencapsulated commercial formulation, Bombex Farumy, for cytotoxic and genotoxic effects in human cell lines. Encapsulated Bombex Farumy showed numerically fewer and smaller significant increases in comet assay measures of DNA damage than the active ingredient. However, this pattern was not confirmed by a direct statistical comparison between formulations, as the only such comparison in this study, between released and encapsulated Bombex Farumy, was not significant at any concentration or endpoint. Therefore, this finding remains tentative and should be confirmed using regulatory compliant assays that can distinguish primary genotoxicity from apoptosis related DNA fragmentation, together with adequately powered direct formulation comparisons. It should not yet be considered evidence that encapsulation provides a safety advantage.

Complementing chromosomal level assessments, the comet assay offers unique advantages for detecting DNA damage at the molecular level. This single cell gel electrophoresis technique demonstrates high sensitivity for early DNA damage detection, enabling researchers to identify genotoxic effects before they manifest as heritable mutations (Gaivao and Sierra, 2014). While the comet assay’s translational relevance for cancer risk prediction may be limited compared to chromosomal damage endpoints, as not all DNA damage detected by comet assay leads to fixed mutations or chromosomal alterations that drive carcinogenesis (Allemang et al., 2021), its ability to detect damage with high sensitivity at the single cell level makes it invaluable for comprehensive genotoxicity evaluation when used in combination with other assessment methods (Brendler-Schwaab et al., 2005).

Across both cell lines and all endpoints, encapsulated Bombex Farumy showed numerically fewer and smaller significant increases relative to control than either the active ingredient or the released formulation. In HL60 cells, encapsulated Bombex Farumy produced no significant increase in DNA percentage in tail or Olive tail moment and showed smaller and less consistent increases in tail length and tail moment than the active ingredient, which remained significant at selected concentrations. In HepG2 cells, encapsulated Bombex Farumy produced fewer significant increases across most parameters than the active ingredient, although Olive tail moment was significantly increased at 1,000 μM (MD: 5.85, p = 0.017). Thus, even in the more resistant hepatic cell line, the encapsulated formulation did not completely eliminate the genotoxic signal at the highest concentration tested. These findings are consistent with, but do not statistically demonstrate, a mitigating effect of microencapsulation. The direct comparison between released and encapsulated Bombex Farumy was not significant at any concentration or endpoint. The occasional higher toxicity of the released formulation than the pure active ingredient may reflect co formulant effects, changes in solubility or bioavailability after methanol extraction, or assay variability. Distinguishing between these possibilities was beyond the scope of this study and requires further testing.

The most significant finding of this research is that cyphenothrin proved to be genotoxic in human cell culture systems. These findings are in contrast with previous research conducted on cyphenothrin active ingredient, which included comprehensive genotoxicity assessments in various animal models. Notably, in the various genotoxicity tests included in its regulatory toxicology package, no genotoxicity of cyphenothrin was observed in living organisms (FSC-Japan, 2023). The discrepancy between our findings and previous animal studies on cyphenothrin active ingredient underscores the complexity of pesticide toxicity and emphasizes the need for thorough evaluation using human cell models alongside traditional animal testing protocols.

Another notable finding was the numerically lower number of significant increases in DNA damage endpoints with encapsulated Bombex Farumy than with the active ingredient across both cell lines. This pattern is consistent with, but does not statistically confirm, a possible mitigating effect of microencapsulation. In HL60 cells, encapsulated Bombex Farumy caused no significant increases in DNA percentage in tail or Olive tail moment, but produced significant, nonmonotonic increases in tail length at 1–150 μM and in tail moment at 1 and 150 μM. In HepG2 cells, fewer significant genotoxic effects were observed with the encapsulated formulation than with the active ingredient across most parameters. However, significant increases in tail moment (MD: 16.64, p = 0.039) and Olive tail moment (MD: 5.85, p = 0.017) occurred at 1,000 μM. Thus, measurable genotoxic effects remained present even in the more resistant hepatic cell line at the highest concentration tested. This pattern is consistent with the controlled release hypothesis, in which the encapsulation matrix may limit cyphenothrin bioavailability and its interaction with cells (Ramadhan Makame et al., 2023; Hack et al., 2012; Kumar et al., 2017; Slattery et al., 2019). We speculate, consistent with the controlled release hypothesis, that encapsulation may modulate cyphenothrin bioavailability by limiting its immediate availability for cellular interaction; however, this interpretation is not directly supported by release profiling data in the present study and should be regarded as hypothesis generating. Empirical measurement of release kinetics under the exposure conditions used here would be required to confirm this mechanism (Butts et al., 2018). Encapsulation may act as a physical or steric barrier that alters exposure of the active material to cells; however, because we did not measure cellular uptake, membrane binding, or internalization, we cannot attribute the observed effects to this mechanism alone (Zhang et al., 2019; Marikar et al., 2022).

The measurable DNA damage associated with the active ingredient across both cell lines, together with the numerically smaller signal observed with encapsulated Bombex Farumy, is consistent with a possible risk reduction effect. However, this interpretation was not statistically confirmed and should therefore remain tentative. If supported by future studies, such a finding could have implications for regulatory approaches that assume encapsulated pesticide formulations are inherently safer. The released formulation showed numerically intermediate toxicity between the active ingredient and the encapsulated form. It also produced significant genotoxic effects across several parameters in HepG2 cells, including DNA percentage in tail, tail length, tail moment, and Olive tail moment at 1,000 μM. This supports successful release of cyphenothrin from the encapsulation matrix and confirms the experimental approach, while also suggesting that the release process may influence toxicological responses. However, the direct statistical comparison between released and encapsulated Bombex Farumy showed no significant differences in any of the 74 concentration by endpoint by cell line comparisons after Holm correction for multiple comparisons within each endpoint by cell line series (smallest Holm-adjusted p = 0.42, HL60 cytotoxicity at 500 μM). Because this was the only formal direct comparison between formulations, the higher HL60 cytotoxicity observed with the released formulation at concentrations ≥200 μM and the higher HepG2 genotoxic signal at 1,000 μM (Table 1) should be regarded as suggestive, not confirmed evidence of a protective effect of encapsulation.

The intermediate toxicity observed with released Bombex Farumy provides additional insight into the possible mechanism. Since co-formulants typically increase toxicity compared with the active ingredient alone (Mesnage et al., 2019), the controlled release properties of the encapsulated formulation may play an important role in the lower genotoxic signal observed in our study. This finding is consistent with the hypothesis that changes in the timing and level of cyphenothrin exposure, without altering its chemical structure, may contribute to the observed reduction in genotoxicity.

Comparative analysis revealed distinct sensitivity patterns between cell lines. HL60 cells demonstrated significantly greater susceptibility to cyphenothrin induced genotoxicity, with significant effects appearing across 50–200 μM concentrations (Table 1). In contrast, HepG2 cells required 10-20 fold higher concentrations (1,000 μM) to induce comparable DNA damage levels. The striking 10-20-fold difference in sensitivity between HL60 and HepG2 cells provides crucial insights into tissue-specific vulnerability to cyphenothrin exposure. HL60 cells demonstrated significant genotoxic responses beginning at 50–100 μM and continuing through the highest tested concentration (200 μM), while HepG2 cells required 1,000 μM to achieve comparable effects. This differential sensitivity likely reflects fundamental differences in cellular defense mechanisms, including metabolic capacity and DNA repair systems, between hematopoietic and hepatic cell types (Gasnier et al., 2009).

The greater resilience of HepG2 cells to cyphenothrin-induced toxicity may relate to their hepatic origin and retained phase II conjugation capacity; however, HepG2 cells are well documented to express phase I cytochrome P450 enzymes at markedly lower levels than primary human hepatocytes (Arzumanian et al., 2021; Stanley and Wolf, 2022; Zhang et al., 2024; Westerink and Schoonen, 2007; Hewitt and Hewitt, 2004; Wilkening et al., 2003) so this explanation should be regarded as tentative and does not rule out other contributors such as differences in proliferation rate, membrane composition, or baseline DNA repair capacity between the 2 cell lines. As the cultures were not supplemented with an exogenous metabolic activation system, any contribution of intrinsic hepatocyte metabolism to this resilience could not be directly tested here. This cellular heterogeneity in pesticide sensitivity aligns with findings from Romero et al. (2015), who identified HepG2 as less sensitive than neuronal cell models when testing pyrethroid mixtures including cyphenothrin (Romero et al., 2015). The hematopoietic system’s heightened vulnerability, represented by HL60 cells, may reflect their increased susceptibility to direct cytotoxic effects due to less active cellular defense mechanisms. Unlike hepatocytes, hematopoietic cells may have limited antioxidant systems and DNA repair mechanisms to counteract pyrethroid induced oxidative stress and direct DNA damage (Barbasz et al., 2020; Montag et al., 2019), which may in part explain their greater sensitivity; however, we did not measure oxidative stress markers or DNA repair activity in the present study, and this remains a speculative interpretation requiring experimental validation. These cell specific patterns have significant implications for human health risk assessment, suggesting that current regulatory approaches may inadequately account for tissue specific vulnerability. Blood forming tissues may represent a more sensitive target for cyphenothrin induced genotoxicity than previously recognized. In addition, HL60 is a promyelocytic leukemia derived cell line with an aberrant karyotype and constitutive MYC amplification, and its sensitivity to genotoxic and cytotoxic insults can vary with differentiation state (Birnie, 1988; Gallagher et al., 1979; Collins, 1987). The pronounced sensitivity of HL60 cells observed may therefore partly reveal leukemic cell intrinsic features such as altered proliferation rate, checkpoint control, or DNA repair capacity but should not considered a generalizable property of normal hematopoietic tissue, and this should be considered when extrapolating our hematopoietic vulnerability interpretation to human risk assessment.

Encapsulated Bombex Farumy produced a non monotonic concentration response pattern in HL60 cells, with significant increases in tail length at 1, 50, 100, and 150 μM, and tail moment at 1 and 150 μM. The disagreement between these parameters–particularly the lack of significant increases in %DNA in tail and Olive tail moment–suggests that tail length and tail moment may detect early structural changes (e.g., DNA relaxation or localized strand breaks) that do not necessarily progress to extensive DNA fragmentation. This pattern highlights the value of using multiple comet assay parameters. However, the absence of consistent dose response across all four parameters also cautions against overinterpreting these individual significant findings as definitive evidence of robust genotoxicity, particularly given that nine of the 33 statistically significant genotoxicity findings reported in Table 1 do not remain significant after a further Holm correction applied across the four correlated comet endpoints tested per chemical x concentration x cell line (Table 1). These borderline findings are concentrated among the encapsulated and released Bombex Farumy tail moment and Olive tail moment results in HL60 (e.g., tail moment at 1 μM, p = 0.049, endpoint adjusted p = 0.147) and the HepG2 active ingredient tail length results at 100–500 μM, and should accordingly be interpreted as suggestive evidence of genotoxicity, in contrast to the active ingredient’s HL60 tail length and tail moment findings at 100–200 μM and the HepG2 findings at 1,000 μM, all of which remain significant after this correction.

Our findings reveal critical gaps in current regulatory frameworks that predominantly assess active ingredient toxicity while inadequately addressing formulation specific effects. The demonstration that encapsulation significantly modifies cyphenothrin’s toxicological profile suggests that regulatory evaluations based solely on active ingredients may misrepresent actual human health risks from commercial pesticide products (EPA. Pesticides, 2005; Cox and Surgan, 2006). Standard genotoxicity testing protocols require modification to include encapsulated forms, as active ingredient testing alone may overestimate risks from encapsulated products while potentially underestimating risks from conventional formulations. We wish to be explicit that the present study is primarily descriptive. The comet assay data identify differences in DNA strand break levels across formulations and cell lines but do not, by themselves, establish the mechanism responsible. Possible explanations including primary genotoxicity *via* direct DNA interaction, oxidative DNA damage secondary to cytotoxic stress, or apoptosis associated DNA fragmentation. This remains to be distinguished through follow-up mechanistic studies.

At concentrations of 100 μM and above, where the active ingredient produced a clear genotoxic signal above background, the mean genotoxic signal for encapsulated Bombex Farumy was numerically 29%–83% lower (median 61%) across the eight endpoint by cell line combinations examined. However, this difference was not directly tested and should therefore be viewed as a descriptive comparison, not a confirmed effect size. Below 100 μM, the active ingredient produced little genuine increase over control, making this ratio unreliable, and it is therefore not reported. Overall, the measurable genotoxic effects of the active ingredient, together with the numerically smaller but statistically unconfirmed signal for encapsulated Bombex Farumy, suggest that encapsulation should be considered a possible harm reduction approach. This distinction is relevant to evidence based regulatory assessment of pesticide formulations while balancing agricultural use with human health protection.

The differential sensitivity patterns between cell types further highlight the need for comprehensive multi cell line assessments in regulatory toxicology. Evaluations based on single cell lines may inadequately capture the complex toxicological profile of pesticide formulations, potentially missing vulnerable target tissues or overestimating safety margins for resistant cell types.

Several methodological limitations must be acknowledged when interpreting our results. The commercial Bombex Farumy formulation contains prallethrin (1.10%) as a secondary active ingredient alongside cyphenothrin (10.70%), introducing complexity in isolating individual component toxicological profiles. Previous genotoxicity studies on prallethrin have reported minimal or no genotoxic effects in various test systems (Dotson et al., 2014). Given its low concentration (1.10% vs. 10.70% cyphenothrin) and reported lower genotoxic potential, prallethrin is unlikely to significantly contribute to the observed DNA damage patterns. However, potential synergistic or antagonistic interactions between cyphenothrin and prallethrin cannot be definitively excluded without component specific testing. Moreover, the methodology employed for releasing the ingredients of the encapsulated formulation, involving methanol extraction, extended vortexing, and centrifugation, represents a potential limitation. The effectiveness of complete extraction without chemical modification cannot be definitively established without analytical verification, and incomplete extraction likely occurred in our study, potentially influencing comparisons between released and encapsulated forms. Additionally, residual methanol effects may have contributed to observed toxicity patterns independently of the active ingredients. Residual methanol content after the 30 min evaporation step was not analytically quantified (e.g., by headspace gas chromatography), and complete removal is therefore not confirmed.

Furthermore, the *in vitro* cell culture systems, while valuable for toxicological investigations, necessarily represent simplified versions of complex *in vivo* biological environments. Cultured cell lines lack three dimensional tissue architecture, cellular heterogeneity, and complete metabolic complexity of intact organs, potentially limiting the translational relevance of our findings (Espos et al., 2024; Yang et al., 2023). This limitation is particularly relevant for cyphenothrin, which undergoes extensive biotransformation *in vivo* (Arslan, 2022; Kaneko et al., 2001). A further limitation is that cytotoxicity and genotoxicity were assessed after a single 1 h exposure; this acute, single timepoint design cannot capture time dependent toxicokinetics, and both shorter and longer exposure windows would be needed to characterize the full dose time response relationship. The released Bombex Farumy concentration is nominal/calculated from the formulation label instead of analytically verified (e.g., by HPLC), and cytotoxicity was assessed using a single membrane integrity readout (propidium iodide exclusion) without an orthogonal metabolic assay; neither could be added within the scope of this concluded project. Propidium iodide exclusion was selected over tetrazolium based assays (MTT/MTS) specifically because HL60 cells grow in suspension, where the wash and formazan solubilization steps of tetrazolium assays are technically unreliable, and PI based enumeration is equally valid for suspension and adherent cultures and specifically recommended for suspension lines in genotoxicity testing protocols (Tice et al., 2000). However, PI membrane integrity staining labels cells with compromised plasma membranes but does not detect early apoptotic cells with intact membranes. Accordingly, some cells classified as viable by PI may have been in early apoptosis, and apoptosis associated DNA fragmentation could have contributed to the comet signal (Alshehade et al., 2024; Vasquez, 2010). Hedgehog/ghost comets were excluded from % tail DNA analysis in line with OECD TG 489; however, a contribution of apoptosis or necrosis related DNA damage to comet positivity cannot be fully excluded without additional apoptosis assays such as Annexin V/PI dual staining or caspase activation analysis.

Important study’s limitations were the single 1 h exposure timepoint, reliance on PI based membrane integrity assessment alone, the released formulation concentration which was not analytically verified, and the possible influence of HL60 specific karyotype and differentiation state. Future research should prioritize empirical release kinetics measurement, apoptosis discriminating assays (e.g., Annexin V/PI or caspase activation), analytical (e.g., HPLC) verification of formulation concentrations, and multi timepoint exposure studies. Such mechanistic studies would enhance understanding of formulation cellular system interactions and guide development of next-generation pesticide technologies with improved safety characteristics. Advanced analytical techniques, including real-time monitoring of encapsulation release patterns and cellular uptake kinetics, would provide crucial mechanistic insights for regulatory translation. In addition, long-term exposure studies representing realistic environmental exposure scenarios are essential for assessing chronic toxicity potential of encapsulated formulations. While our acute exposure model provides valuable toxicological information, chronic low dose exposure patterns may reveal different toxicological profiles requiring distinct risk assessment approaches.

## 5 Conclusion

This study provides preliminary, hypothesis generating evidence that encapsulated Bombex Farumy is associated with numerically fewer and smaller significant increases in comet assay measures of DNA damage than cyphenothrin in human cell lines. If confirmed, this pattern would be consistent with a reduction in genotoxic potential following encapsulation, although not complete elimination. However, the only direct statistical comparison between released and encapsulated Bombex Farumy was not significant at any concentration or endpoint after correction for multiple comparisons. In addition, the actual cyphenothrin concentration in the released formulation and its residual methanol content were not analytically verified. Therefore, a true mitigating effect of microencapsulation cannot be established from the present data and should remain a hypothesis for future adequately powered, formulation focused studies. The observed pattern is compatible with, but does not directly demonstrate, controlled release or reduced bioavailability, as no release kinetics, cellular uptake, or membrane binding data were obtained. It is also unclear whether the observed DNA damage reflects primary genotoxicity, apoptosis related DNA fragmentation, or both. This requires confirmation using regulatory compliant assay batteries that include apoptosis markers. Despite these limitations, the findings provide a basis for formulation specific toxicological assessment and highlight a potential gap in regulatory approaches that focus primarily on active ingredients. These findings should justify comprehensive follow-up investigations using validated OECD protocols, ultimately contributing to evidence based pesticide safety assessment. The numerically consistent pattern, fewer and smaller significant deviations from control for encapsulated Bombex Farumy across multiple genotoxicity parameters and two distinct cell lines, adds some internal consistency to these observations, although it does not substitute for a significant direct formulation contrast. The identification of pronounced genotoxic sensitivity in the HL60 leukocyte lineage model used here should be considered alongside this cell line’s transformed, leukemia derived karyotype and differentiation state dependent sensitivity, and should warrant further investigation of its implications for health protection strategies, although it should not be generalized to normal hematopoietic tissue. Our findings contribute to a growing body of evidence that formulation technology may modulate pesticide toxicity, and provide a rationale for further work toward safer cyphenothrin based products, although this specific study did not statistically confirm a difference between formulations. The persistence of measurable genotoxic effects in the encapsulated formulation itself, regardless of any comparison with other formulations, emphasizes the need for continued vigilance in pesticide risk assessment and management.

## Data Availability

The raw data supporting the conclusions of this article will be made available by the authors, without undue reservation.
