# Supplementary material for: Cytotoxicity and genotoxicity of cyphenothrin active ingredient compared with its microencapsulated commercial formulation in human cell lines: an in vitro study
Source: Front Toxicol. 2026 Oct 23;8:1932062. doi: 10.3389/ftox.2026.1932062 (PMC13740607; doi:10.3389/ftox.2026.1932062)
Supplement: Supplementary file 1 [file Table1.docx]

Supplementary Table S1. Summary statistics for cytotoxicity and genotoxicity endpoints, by chemical, cell line, and concentration, versus untreated control (one-way ANOVA with Dunnett's post-hoc test).

| Cell line | Assay | Endpoint | Chemical | Conc. (µM) | Mean diff (SE) | p (raw) | p (endpoint-adj.) |
| --- | --- | --- | --- | --- | --- | --- | --- |
| HL60 | cytotoxicity | viability_pct | Cyphenothrin Active Ingredient | 0.10 | -4.97 (4.032) | 0.752 | 0.752 |
| HL60 | cytotoxicity | viability_pct | Cyphenothrin Active Ingredient | 1.00 | -7.83 (3.681) | 0.229 | 0.229 |
| HL60 | cytotoxicity | viability_pct | Cyphenothrin Active Ingredient | 10.00 | -5.50 (3.681) | 0.572 | 0.572 |
| HL60 | cytotoxicity | viability_pct | Cyphenothrin Active Ingredient | 100.00 | -4.17 (3.681) | 0.816 | 0.816 |
| HL60 | cytotoxicity | viability_pct | Cyphenothrin Active Ingredient | 200.00 | -19.50 (3.681) | <0.001 | <0.001 |
| HL60 | cytotoxicity | viability_pct | Cyphenothrin Active Ingredient | 300.00 | -32.50 (3.681) | <0.001 | <0.001 |
| HL60 | cytotoxicity | viability_pct | Cyphenothrin Active Ingredient | 500.00 | -35.50 (3.681) | <0.001 | <0.001 |
| HL60 | cytotoxicity | viability_pct | Cyphenothrin Active Ingredient | 750.00 | -73.17 (3.681) | <0.001 | <0.001 |
| HL60 | cytotoxicity | viability_pct | Cyphenothrin Active Ingredient | 1,000.00 | -94.50 (3.681) | <0.001 | <0.001 |
| HL60 | cytotoxicity | viability_pct | Encapsulated Bombex Farumy | 0.10 | -3.83 (4.145) | 0.920 | 0.920 |
| HL60 | cytotoxicity | viability_pct | Encapsulated Bombex Farumy | 1.00 | -3.83 (4.145) | 0.920 | 0.920 |
| HL60 | cytotoxicity | viability_pct | Encapsulated Bombex Farumy | 10.00 | -6.50 (4.145) | 0.516 | 0.516 |
| HL60 | cytotoxicity | viability_pct | Encapsulated Bombex Farumy | 100.00 | -8.50 (4.145) | 0.254 | 0.254 |
| HL60 | cytotoxicity | viability_pct | Encapsulated Bombex Farumy | 200.00 | -14.83 (4.145) | 0.013 | 0.013 |
| HL60 | cytotoxicity | viability_pct | Encapsulated Bombex Farumy | 300.00 | -16.83 (4.145) | 0.004 | 0.004 |
| HL60 | cytotoxicity | viability_pct | Encapsulated Bombex Farumy | 500.00 | -27.17 (4.145) | <0.001 | <0.001 |
| HL60 | cytotoxicity | viability_pct | Encapsulated Bombex Farumy | 750.00 | -37.50 (4.145) | <0.001 | <0.001 |
| HL60 | cytotoxicity | viability_pct | Encapsulated Bombex Farumy | 1,000.00 | -52.50 (4.145) | <0.001 | <0.001 |
| HL60 | cytotoxicity | viability_pct | Released Bombex Farumy | 0.10 | -5.83 (5.056) | 0.807 | 0.807 |
| HL60 | cytotoxicity | viability_pct | Released Bombex Farumy | 1.00 | -3.50 (5.056) | 0.985 | 0.985 |
| HL60 | cytotoxicity | viability_pct | Released Bombex Farumy | 10.00 | -4.50 (5.056) | 0.938 | 0.938 |
| HL60 | cytotoxicity | viability_pct | Released Bombex Farumy | 100.00 | -11.83 (5.056) | 0.164 | 0.164 |
| HL60 | cytotoxicity | viability_pct | Released Bombex Farumy | 200.00 | -7.83 (5.056) | 0.542 | 0.542 |
| HL60 | cytotoxicity | viability_pct | Released Bombex Farumy | 300.00 | -12.50 (5.539) | 0.189 | 0.189 |
| HL60 | cytotoxicity | viability_pct | Released Bombex Farumy | 500.00 | -16.50 (5.056) | 0.028 | 0.028 |
| HL60 | cytotoxicity | viability_pct | Released Bombex Farumy | 750.00 | -25.00 (5.539) | 0.002 | 0.002 |
| HL60 | cytotoxicity | viability_pct | Released Bombex Farumy | 1,000.00 | -41.83 (5.056) | <0.001 | <0.001 |
| HL60 | genotoxicity | DNA_in_Ta | Cyphenothrin Active Ingredient | 0.10 | 5.17 (6.805) | 0.971 | 1.000 |
| HL60 | genotoxicity | DNA_in_Ta | Cyphenothrin Active Ingredient | 1.00 | 2.67 (6.805) | 1.000 | 1.000 |
| HL60 | genotoxicity | DNA_in_Ta | Cyphenothrin Active Ingredient | 10.00 | 2.67 (6.086) | 0.999 | 1.000 |
| HL60 | genotoxicity | DNA_in_Ta | Cyphenothrin Active Ingredient | 50.00 | 7.67 (6.805) | 0.828 | 1.000 |
| HL60 | genotoxicity | DNA_in_Ta | Cyphenothrin Active Ingredient | 100.00 | 26.17 (6.805) | 0.013 | 0.025 |
| HL60 | genotoxicity | DNA_in_Ta | Cyphenothrin Active Ingredient | 150.00 | 18.67 (6.805) | 0.092 | 0.092 |
| HL60 | genotoxicity | DNA_in_Ta | Cyphenothrin Active Ingredient | 200.00 | 27.67 (6.086) | 0.004 | 0.005 |
| HL60 | genotoxicity | DNA_in_Ta | Encapsulated Bombex Farumy | 0.10 | -12.33 (5.747) | 0.248 | 0.992 |
| HL60 | genotoxicity | DNA_in_Ta | Encapsulated Bombex Farumy | 1.00 | 11.67 (5.747) | 0.296 | 0.592 |
| HL60 | genotoxicity | DNA_in_Ta | Encapsulated Bombex Farumy | 10.00 | -4.33 (5.747) | 0.972 | 1.000 |
| HL60 | genotoxicity | DNA_in_Ta | Encapsulated Bombex Farumy | 50.00 | 5.17 (5.747) | 0.932 | 1.000 |
| HL60 | genotoxicity | DNA_in_Ta | Encapsulated Bombex Farumy | 100.00 | 10.00 (5.140) | 0.335 | 0.669 |
| HL60 | genotoxicity | DNA_in_Ta | Encapsulated Bombex Farumy | 150.00 | 9.67 (5.747) | 0.478 | 0.624 |
| HL60 | genotoxicity | DNA_in_Ta | Encapsulated Bombex Farumy | 200.00 | 9.00 (5.140) | 0.438 | 0.737 |
| HL60 | genotoxicity | DNA_in_Ta | Released Bombex Farumy | 0.10 | -0.83 (7.318) | 1.000 | 1.000 |
| HL60 | genotoxicity | DNA_in_Ta | Released Bombex Farumy | 1.00 | -0.33 (7.318) | 1.000 | 1.000 |
| HL60 | genotoxicity | DNA_in_Ta | Released Bombex Farumy | 10.00 | -2.33 (7.318) | 1.000 | 1.000 |
| HL60 | genotoxicity | DNA_in_Ta | Released Bombex Farumy | 50.00 | 12.67 (7.318) | 0.465 | 0.579 |
| HL60 | genotoxicity | DNA_in_Ta | Released Bombex Farumy | 100.00 | 17.17 (7.318) | 0.195 | 0.195 |
| HL60 | genotoxicity | DNA_in_Ta | Released Bombex Farumy | 150.00 | 4.67 (7.318) | 0.989 | 1.000 |
| HL60 | genotoxicity | DNA_in_Ta | Released Bombex Farumy | 200.00 | 8.17 (7.318) | 0.840 | 1.000 |
| HL60 | genotoxicity | Olive_moment | Cyphenothrin Active Ingredient | 0.10 | 1.67 (2.576) | 0.988 | 1.000 |
| HL60 | genotoxicity | Olive_moment | Cyphenothrin Active Ingredient | 1.00 | 1.17 (2.576) | 0.999 | 1.000 |
| HL60 | genotoxicity | Olive_moment | Cyphenothrin Active Ingredient | 10.00 | 1.67 (2.304) | 0.977 | 1.000 |
| HL60 | genotoxicity | Olive_moment | Cyphenothrin Active Ingredient | 50.00 | 2.67 (2.576) | 0.875 | 1.000 |
| HL60 | genotoxicity | Olive_moment | Cyphenothrin Active Ingredient | 100.00 | 8.17 (2.576) | 0.043 | 0.043 |
| HL60 | genotoxicity | Olive_moment | Cyphenothrin Active Ingredient | 150.00 | 8.17 (2.576) | 0.043 | 0.086 |
| HL60 | genotoxicity | Olive_moment | Cyphenothrin Active Ingredient | 200.00 | 11.00 (2.304) | 0.002 | 0.005 |
| HL60 | genotoxicity | Olive_moment | Encapsulated Bombex Farumy | 0.10 | -1.83 (1.588) | 0.812 | 1.000 |
| HL60 | genotoxicity | Olive_moment | Encapsulated Bombex Farumy | 1.00 | 2.17 (1.588) | 0.682 | 0.682 |
| HL60 | genotoxicity | Olive_moment | Encapsulated Bombex Farumy | 10.00 | -0.33 (1.588) | 1.000 | 1.000 |
| HL60 | genotoxicity | Olive_moment | Encapsulated Bombex Farumy | 50.00 | 2.17 (1.588) | 0.682 | 1.000 |
| HL60 | genotoxicity | Olive_moment | Encapsulated Bombex Farumy | 100.00 | 2.67 (1.420) | 0.369 | 0.669 |
| HL60 | genotoxicity | Olive_moment | Encapsulated Bombex Farumy | 150.00 | 3.17 (1.588) | 0.312 | 0.624 |
| HL60 | genotoxicity | Olive_moment | Encapsulated Bombex Farumy | 200.00 | 2.67 (1.420) | 0.368 | 0.737 |
| HL60 | genotoxicity | Olive_moment | Released Bombex Farumy | 0.10 | 0.17 (2.601) | 1.000 | 1.000 |
| HL60 | genotoxicity | Olive_moment | Released Bombex Farumy | 1.00 | 0.17 (2.601) | 1.000 | 1.000 |
| HL60 | genotoxicity | Olive_moment | Released Bombex Farumy | 10.00 | 0.17 (2.601) | 1.000 | 1.000 |
| HL60 | genotoxicity | Olive_moment | Released Bombex Farumy | 50.00 | 5.67 (2.601) | 0.251 | 0.579 |
| HL60 | genotoxicity | Olive_moment | Released Bombex Farumy | 100.00 | 7.67 (2.601) | 0.074 | 0.149 |
| HL60 | genotoxicity | Olive_moment | Released Bombex Farumy | 150.00 | 1.17 (2.601) | 0.999 | 1.000 |
| HL60 | genotoxicity | Olive_moment | Released Bombex Farumy | 200.00 | 2.67 (2.601) | 0.885 | 1.000 |
| HL60 | genotoxicity | Tail_Length | Cyphenothrin Active Ingredient | 0.10 | 7.00 (9.753) | 0.978 | 1.000 |
| HL60 | genotoxicity | Tail_Length | Cyphenothrin Active Ingredient | 1.00 | 13.00 (9.753) | 0.702 | 1.000 |
| HL60 | genotoxicity | Tail_Length | Cyphenothrin Active Ingredient | 10.00 | 16.67 (8.723) | 0.352 | 1.000 |
| HL60 | genotoxicity | Tail_Length | Cyphenothrin Active Ingredient | 50.00 | 24.50 (9.753) | 0.137 | 0.548 |
| HL60 | genotoxicity | Tail_Length | Cyphenothrin Active Ingredient | 100.00 | 57.00 (9.753) | <0.001 | 0.002 |
| HL60 | genotoxicity | Tail_Length | Cyphenothrin Active Ingredient | 150.00 | 62.00 (9.753) | <0.001 | <0.001 |
| HL60 | genotoxicity | Tail_Length | Cyphenothrin Active Ingredient | 200.00 | 51.33 (8.723) | <0.001 | 0.001 |
| HL60 | genotoxicity | Tail_Length | Encapsulated Bombex Farumy | 0.10 | -10.00 (6.486) | 0.566 | 1.000 |
| HL60 | genotoxicity | Tail_Length | Encapsulated Bombex Farumy | 1.00 | 33.50 (6.486) | 0.001 | 0.005 |
| HL60 | genotoxicity | Tail_Length | Encapsulated Bombex Farumy | 10.00 | -0.50 (6.486) | 1.000 | 1.000 |
| HL60 | genotoxicity | Tail_Length | Encapsulated Bombex Farumy | 50.00 | 28.00 (6.486) | 0.005 | 0.021 |
| HL60 | genotoxicity | Tail_Length | Encapsulated Bombex Farumy | 100.00 | 21.33 (5.802) | 0.017 | 0.069 |
| HL60 | genotoxicity | Tail_Length | Encapsulated Bombex Farumy | 150.00 | 31.00 (6.486) | 0.002 | 0.009 |
| HL60 | genotoxicity | Tail_Length | Encapsulated Bombex Farumy | 200.00 | 16.33 (5.802) | 0.081 | 0.326 |
| HL60 | genotoxicity | Tail_Length | Released Bombex Farumy | 0.10 | 1.00 (6.700) | 1.000 | 1.000 |
| HL60 | genotoxicity | Tail_Length | Released Bombex Farumy | 1.00 | 9.00 (6.700) | 0.705 | 1.000 |
| HL60 | genotoxicity | Tail_Length | Released Bombex Farumy | 10.00 | 5.00 (6.700) | 0.974 | 1.000 |
| HL60 | genotoxicity | Tail_Length | Released Bombex Farumy | 50.00 | 34.00 (6.700) | 0.002 | 0.010 |
| HL60 | genotoxicity | Tail_Length | Released Bombex Farumy | 100.00 | 46.00 (6.700) | <0.001 | 0.001 |
| HL60 | genotoxicity | Tail_Length | Released Bombex Farumy | 150.00 | 18.50 (6.700) | 0.101 | 0.403 |
| HL60 | genotoxicity | Tail_Length | Released Bombex Farumy | 200.00 | 28.00 (6.700) | 0.010 | 0.039 |
| HL60 | genotoxicity | Tail_moment | Cyphenothrin Active Ingredient | 0.10 | 2.33 (5.808) | 0.999 | 1.000 |
| HL60 | genotoxicity | Tail_moment | Cyphenothrin Active Ingredient | 1.00 | 3.83 (5.808) | 0.987 | 1.000 |
| HL60 | genotoxicity | Tail_moment | Cyphenothrin Active Ingredient | 10.00 | 4.00 (5.195) | 0.968 | 1.000 |
| HL60 | genotoxicity | Tail_moment | Cyphenothrin Active Ingredient | 50.00 | 8.33 (5.808) | 0.635 | 1.000 |
| HL60 | genotoxicity | Tail_moment | Cyphenothrin Active Ingredient | 100.00 | 30.83 (5.808) | <0.001 | 0.003 |
| HL60 | genotoxicity | Tail_moment | Cyphenothrin Active Ingredient | 150.00 | 27.83 (5.808) | 0.002 | 0.007 |
| HL60 | genotoxicity | Tail_moment | Cyphenothrin Active Ingredient | 200.00 | 30.67 (5.195) | <0.001 | 0.001 |
| HL60 | genotoxicity | Tail_moment | Encapsulated Bombex Farumy | 0.10 | -2.67 (3.495) | 0.970 | 1.000 |
| HL60 | genotoxicity | Tail_moment | Encapsulated Bombex Farumy | 1.00 | 10.83 (3.495) | 0.049 | 0.147 |
| HL60 | genotoxicity | Tail_moment | Encapsulated Bombex Farumy | 10.00 | -1.67 (3.495) | 0.998 | 1.000 |
| HL60 | genotoxicity | Tail_moment | Encapsulated Bombex Farumy | 50.00 | 7.33 (3.495) | 0.267 | 0.800 |
| HL60 | genotoxicity | Tail_moment | Encapsulated Bombex Farumy | 100.00 | 7.33 (3.126) | 0.181 | 0.542 |
| HL60 | genotoxicity | Tail_moment | Encapsulated Bombex Farumy | 150.00 | 11.33 (3.495) | 0.038 | 0.113 |
| HL60 | genotoxicity | Tail_moment | Encapsulated Bombex Farumy | 200.00 | 7.67 (3.126) | 0.151 | 0.454 |
| HL60 | genotoxicity | Tail_moment | Released Bombex Farumy | 0.10 | -0.67 (6.089) | 1.000 | 1.000 |
| HL60 | genotoxicity | Tail_moment | Released Bombex Farumy | 1.00 | 0.83 (6.089) | 1.000 | 1.000 |
| HL60 | genotoxicity | Tail_moment | Released Bombex Farumy | 10.00 | -0.17 (6.089) | 1.000 | 1.000 |
| HL60 | genotoxicity | Tail_moment | Released Bombex Farumy | 50.00 | 14.33 (6.089) | 0.193 | 0.579 |
| HL60 | genotoxicity | Tail_moment | Released Bombex Farumy | 100.00 | 20.83 (6.089) | 0.034 | 0.101 |
| HL60 | genotoxicity | Tail_moment | Released Bombex Farumy | 150.00 | 4.33 (6.089) | 0.980 | 1.000 |
| HL60 | genotoxicity | Tail_moment | Released Bombex Farumy | 200.00 | 7.83 (6.089) | 0.741 | 1.000 |
| HepG2 | cytotoxicity | viability_pct | Cyphenothrin Active Ingredient | 0.10 | 1.33 (2.559) | 1.000 | 1.000 |
| HepG2 | cytotoxicity | viability_pct | Cyphenothrin Active Ingredient | 1.00 | 0.83 (2.559) | 1.000 | 1.000 |
| HepG2 | cytotoxicity | viability_pct | Cyphenothrin Active Ingredient | 10.00 | 4.33 (2.559) | 0.585 | 0.585 |
| HepG2 | cytotoxicity | viability_pct | Cyphenothrin Active Ingredient | 100.00 | -0.17 (2.559) | 1.000 | 1.000 |
| HepG2 | cytotoxicity | viability_pct | Cyphenothrin Active Ingredient | 200.00 | 0.33 (2.559) | 1.000 | 1.000 |
| HepG2 | cytotoxicity | viability_pct | Cyphenothrin Active Ingredient | 500.00 | -4.17 (2.559) | 0.629 | 0.629 |
| HepG2 | cytotoxicity | viability_pct | Cyphenothrin Active Ingredient | 1,000.00 | -1.67 (2.559) | 0.998 | 0.998 |
| HepG2 | cytotoxicity | viability_pct | Cyphenothrin Active Ingredient | 2,000.00 | -8.17 (2.559) | 0.050 | 0.050 |
| HepG2 | cytotoxicity | viability_pct | Cyphenothrin Active Ingredient | 4,000.00 | -20.67 (2.559) | <0.001 | <0.001 |
| HepG2 | cytotoxicity | viability_pct | Encapsulated Bombex Farumy | 0.10 | 7.33 (2.439) | 0.071 | 0.071 |
| HepG2 | cytotoxicity | viability_pct | Encapsulated Bombex Farumy | 1.00 | -1.67 (2.439) | 0.996 | 0.996 |
| HepG2 | cytotoxicity | viability_pct | Encapsulated Bombex Farumy | 10.00 | 0.33 (2.439) | 1.000 | 1.000 |
| HepG2 | cytotoxicity | viability_pct | Encapsulated Bombex Farumy | 100.00 | -0.17 (2.439) | 1.000 | 1.000 |
| HepG2 | cytotoxicity | viability_pct | Encapsulated Bombex Farumy | 200.00 | -2.67 (2.439) | 0.927 | 0.927 |
| HepG2 | cytotoxicity | viability_pct | Encapsulated Bombex Farumy | 500.00 | -2.17 (2.439) | 0.978 | 0.978 |
| HepG2 | cytotoxicity | viability_pct | Encapsulated Bombex Farumy | 1,000.00 | -8.67 (2.439) | 0.025 | 0.025 |
| HepG2 | cytotoxicity | viability_pct | Encapsulated Bombex Farumy | 2,000.00 | -8.17 (2.439) | 0.037 | 0.037 |
| HepG2 | cytotoxicity | viability_pct | Encapsulated Bombex Farumy | 4,000.00 | -21.67 (2.439) | <0.001 | <0.001 |
| HepG2 | cytotoxicity | viability_pct | Released Bombex Farumy | 0.10 | 2.83 (3.437) | 0.987 | 0.987 |
| HepG2 | cytotoxicity | viability_pct | Released Bombex Farumy | 1.00 | -3.17 (3.437) | 0.972 | 0.972 |
| HepG2 | cytotoxicity | viability_pct | Released Bombex Farumy | 10.00 | 1.83 (3.437) | 1.000 | 1.000 |
| HepG2 | cytotoxicity | viability_pct | Released Bombex Farumy | 100.00 | -4.67 (3.437) | 0.802 | 0.802 |
| HepG2 | cytotoxicity | viability_pct | Released Bombex Farumy | 200.00 | -8.17 (3.437) | 0.220 | 0.220 |
| HepG2 | cytotoxicity | viability_pct | Released Bombex Farumy | 500.00 | -2.17 (3.437) | 0.998 | 0.998 |
| HepG2 | cytotoxicity | viability_pct | Released Bombex Farumy | 1,000.00 | -6.67 (3.437) | 0.429 | 0.429 |
| HepG2 | cytotoxicity | viability_pct | Released Bombex Farumy | 2,000.00 | -16.17 (3.437) | 0.003 | 0.003 |
| HepG2 | cytotoxicity | viability_pct | Released Bombex Farumy | 4,000.00 | -23.17 (3.437) | <0.001 | <0.001 |
| HepG2 | genotoxicity | DNA_in_Ta | Cyphenothrin Active Ingredient | 0.01 | 7.64 (7.130) | 0.913 | 1.000 |
| HepG2 | genotoxicity | DNA_in_Ta | Cyphenothrin Active Ingredient | 0.10 | 2.30 (5.330) | 1.000 | 1.000 |
| HepG2 | genotoxicity | DNA_in_Ta | Cyphenothrin Active Ingredient | 1.00 | 10.02 (5.330) | 0.397 | 1.000 |
| HepG2 | genotoxicity | DNA_in_Ta | Cyphenothrin Active Ingredient | 10.00 | -0.27 (4.891) | 1.000 | 1.000 |
| HepG2 | genotoxicity | DNA_in_Ta | Cyphenothrin Active Ingredient | 100.00 | 13.01 (5.330) | 0.138 | 0.202 |
| HepG2 | genotoxicity | DNA_in_Ta | Cyphenothrin Active Ingredient | 500.00 | 13.65 (5.330) | 0.106 | 0.212 |
| HepG2 | genotoxicity | DNA_in_Ta | Cyphenothrin Active Ingredient | 1,000.00 | 28.39 (5.330) | <0.001 | <0.001 |
| HepG2 | genotoxicity | DNA_in_Ta | Encapsulated Bombex Farumy | 0.01 | 12.83 (7.671) | 0.538 | 1.000 |
| HepG2 | genotoxicity | DNA_in_Ta | Encapsulated Bombex Farumy | 0.10 | 3.88 (5.262) | 0.989 | 1.000 |
| HepG2 | genotoxicity | DNA_in_Ta | Encapsulated Bombex Farumy | 1.00 | 8.35 (5.734) | 0.693 | 1.000 |
| HepG2 | genotoxicity | DNA_in_Ta | Encapsulated Bombex Farumy | 10.00 | 0.21 (6.445) | 1.000 | 1.000 |
| HepG2 | genotoxicity | DNA_in_Ta | Encapsulated Bombex Farumy | 100.00 | 2.21 (5.262) | 1.000 | 1.000 |
| HepG2 | genotoxicity | DNA_in_Ta | Encapsulated Bombex Farumy | 500.00 | 8.63 (5.734) | 0.658 | 1.000 |
| HepG2 | genotoxicity | DNA_in_Ta | Encapsulated Bombex Farumy | 1,000.00 | 15.50 (5.734) | 0.077 | 0.153 |
| HepG2 | genotoxicity | DNA_in_Ta | Released Bombex Farumy | 0.01 | 3.66 (9.337) | 1.000 | 1.000 |
| HepG2 | genotoxicity | DNA_in_Ta | Released Bombex Farumy | 0.10 | 7.32 (7.845) | 0.958 | 1.000 |
| HepG2 | genotoxicity | DNA_in_Ta | Released Bombex Farumy | 1.00 | 1.28 (7.845) | 1.000 | 1.000 |
| HepG2 | genotoxicity | DNA_in_Ta | Released Bombex Farumy | 10.00 | 13.51 (7.845) | 0.508 | 0.940 |
| HepG2 | genotoxicity | DNA_in_Ta | Released Bombex Farumy | 100.00 | 1.99 (6.980) | 1.000 | 1.000 |
| HepG2 | genotoxicity | DNA_in_Ta | Released Bombex Farumy | 500.00 | 11.17 (6.980) | 0.595 | 1.000 |
| HepG2 | genotoxicity | DNA_in_Ta | Released Bombex Farumy | 1,000.00 | 35.26 (6.980) | <0.001 | <0.001 |
| HepG2 | genotoxicity | Olive_moment | Cyphenothrin Active Ingredient | 0.01 | 1.05 (2.355) | 1.000 | 1.000 |
| HepG2 | genotoxicity | Olive_moment | Cyphenothrin Active Ingredient | 0.10 | 0.25 (1.760) | 1.000 | 1.000 |
| HepG2 | genotoxicity | Olive_moment | Cyphenothrin Active Ingredient | 1.00 | 3.26 (1.760) | 0.415 | 1.000 |
| HepG2 | genotoxicity | Olive_moment | Cyphenothrin Active Ingredient | 10.00 | -0.28 (1.615) | 1.000 | 1.000 |
| HepG2 | genotoxicity | Olive_moment | Cyphenothrin Active Ingredient | 100.00 | 4.86 (1.760) | 0.067 | 0.202 |
| HepG2 | genotoxicity | Olive_moment | Cyphenothrin Active Ingredient | 500.00 | 4.34 (1.760) | 0.131 | 0.212 |
| HepG2 | genotoxicity | Olive_moment | Cyphenothrin Active Ingredient | 1,000.00 | 10.44 (1.760) | <0.001 | <0.001 |
| HepG2 | genotoxicity | Olive_moment | Encapsulated Bombex Farumy | 0.01 | 3.08 (2.364) | 0.794 | 1.000 |
| HepG2 | genotoxicity | Olive_moment | Encapsulated Bombex Farumy | 0.10 | 0.80 (1.621) | 0.999 | 1.000 |
| HepG2 | genotoxicity | Olive_moment | Encapsulated Bombex Farumy | 1.00 | 2.30 (1.767) | 0.796 | 1.000 |
| HepG2 | genotoxicity | Olive_moment | Encapsulated Bombex Farumy | 10.00 | -0.18 (1.986) | 1.000 | 1.000 |
| HepG2 | genotoxicity | Olive_moment | Encapsulated Bombex Farumy | 100.00 | 0.92 (1.621) | 0.998 | 1.000 |
| HepG2 | genotoxicity | Olive_moment | Encapsulated Bombex Farumy | 500.00 | 3.09 (1.767) | 0.485 | 1.000 |
| HepG2 | genotoxicity | Olive_moment | Encapsulated Bombex Farumy | 1,000.00 | 5.85 (1.767) | 0.017 | 0.067 |
| HepG2 | genotoxicity | Olive_moment | Released Bombex Farumy | 0.01 | 2.38 (3.670) | 0.995 | 1.000 |
| HepG2 | genotoxicity | Olive_moment | Released Bombex Farumy | 0.10 | 4.76 (3.083) | 0.635 | 1.000 |
| HepG2 | genotoxicity | Olive_moment | Released Bombex Farumy | 1.00 | 2.24 (3.083) | 0.990 | 1.000 |
| HepG2 | genotoxicity | Olive_moment | Released Bombex Farumy | 10.00 | 6.75 (3.083) | 0.235 | 0.940 |
| HepG2 | genotoxicity | Olive_moment | Released Bombex Farumy | 100.00 | 2.49 (2.743) | 0.964 | 1.000 |
| HepG2 | genotoxicity | Olive_moment | Released Bombex Farumy | 500.00 | 4.70 (2.743) | 0.513 | 1.000 |
| HepG2 | genotoxicity | Olive_moment | Released Bombex Farumy | 1,000.00 | 15.62 (2.743) | <0.001 | <0.001 |
| HepG2 | genotoxicity | Tail_Length | Cyphenothrin Active Ingredient | 0.01 | 5.24 (10.776) | 0.999 | 1.000 |
| HepG2 | genotoxicity | Tail_Length | Cyphenothrin Active Ingredient | 0.10 | 9.47 (8.056) | 0.866 | 1.000 |
| HepG2 | genotoxicity | Tail_Length | Cyphenothrin Active Ingredient | 1.00 | 16.29 (8.056) | 0.313 | 1.000 |
| HepG2 | genotoxicity | Tail_Length | Cyphenothrin Active Ingredient | 10.00 | 3.84 (7.393) | 0.999 | 1.000 |
| HepG2 | genotoxicity | Tail_Length | Cyphenothrin Active Ingredient | 100.00 | 23.54 (8.056) | 0.045 | 0.181 |
| HepG2 | genotoxicity | Tail_Length | Cyphenothrin Active Ingredient | 500.00 | 26.22 (8.056) | 0.020 | 0.078 |
| HepG2 | genotoxicity | Tail_Length | Cyphenothrin Active Ingredient | 1,000.00 | 38.00 (8.056) | <0.001 | <0.001 |
| HepG2 | genotoxicity | Tail_Length | Encapsulated Bombex Farumy | 0.01 | 29.86 (11.550) | 0.100 | 0.401 |
| HepG2 | genotoxicity | Tail_Length | Encapsulated Bombex Farumy | 0.10 | 8.14 (7.923) | 0.930 | 1.000 |
| HepG2 | genotoxicity | Tail_Length | Encapsulated Bombex Farumy | 1.00 | 11.59 (8.634) | 0.770 | 1.000 |
| HepG2 | genotoxicity | Tail_Length | Encapsulated Bombex Farumy | 10.00 | -3.12 (9.704) | 1.000 | 1.000 |
| HepG2 | genotoxicity | Tail_Length | Encapsulated Bombex Farumy | 100.00 | 9.44 (7.923) | 0.858 | 1.000 |
| HepG2 | genotoxicity | Tail_Length | Encapsulated Bombex Farumy | 500.00 | 16.34 (8.634) | 0.389 | 1.000 |
| HepG2 | genotoxicity | Tail_Length | Encapsulated Bombex Farumy | 1,000.00 | 22.65 (8.634) | 0.092 | 0.153 |
| HepG2 | genotoxicity | Tail_Length | Released Bombex Farumy | 0.01 | 8.37 (10.894) | 0.987 | 1.000 |
| HepG2 | genotoxicity | Tail_Length | Released Bombex Farumy | 0.10 | 16.74 (9.152) | 0.436 | 1.000 |
| HepG2 | genotoxicity | Tail_Length | Released Bombex Farumy | 1.00 | 6.39 (9.152) | 0.993 | 1.000 |
| HepG2 | genotoxicity | Tail_Length | Released Bombex Farumy | 10.00 | 19.16 (9.152) | 0.281 | 0.940 |
| HepG2 | genotoxicity | Tail_Length | Released Bombex Farumy | 100.00 | 11.06 (8.143) | 0.763 | 1.000 |
| HepG2 | genotoxicity | Tail_Length | Released Bombex Farumy | 500.00 | 19.37 (8.143) | 0.162 | 0.650 |
| HepG2 | genotoxicity | Tail_Length | Released Bombex Farumy | 1,000.00 | 30.11 (8.143) | 0.007 | 0.007 |
| HepG2 | genotoxicity | Tail_moment | Cyphenothrin Active Ingredient | 0.01 | 1.87 (6.825) | 1.000 | 1.000 |
| HepG2 | genotoxicity | Tail_moment | Cyphenothrin Active Ingredient | 0.10 | 1.90 (5.102) | 1.000 | 1.000 |
| HepG2 | genotoxicity | Tail_moment | Cyphenothrin Active Ingredient | 1.00 | 9.88 (5.102) | 0.361 | 1.000 |
| HepG2 | genotoxicity | Tail_moment | Cyphenothrin Active Ingredient | 10.00 | -1.08 (4.682) | 1.000 | 1.000 |
| HepG2 | genotoxicity | Tail_moment | Cyphenothrin Active Ingredient | 100.00 | 13.85 (5.102) | 0.075 | 0.202 |
| HepG2 | genotoxicity | Tail_moment | Cyphenothrin Active Ingredient | 500.00 | 13.99 (5.102) | 0.070 | 0.209 |
| HepG2 | genotoxicity | Tail_moment | Cyphenothrin Active Ingredient | 1,000.00 | 29.63 (5.102) | <0.001 | <0.001 |
| HepG2 | genotoxicity | Tail_moment | Encapsulated Bombex Farumy | 0.01 | 11.74 (7.470) | 0.611 | 1.000 |
| HepG2 | genotoxicity | Tail_moment | Encapsulated Bombex Farumy | 0.10 | 2.29 (5.124) | 1.000 | 1.000 |
| HepG2 | genotoxicity | Tail_moment | Encapsulated Bombex Farumy | 1.00 | 6.32 (5.584) | 0.887 | 1.000 |
| HepG2 | genotoxicity | Tail_moment | Encapsulated Bombex Farumy | 10.00 | -1.24 (6.276) | 1.000 | 1.000 |
| HepG2 | genotoxicity | Tail_moment | Encapsulated Bombex Farumy | 100.00 | 2.99 (5.124) | 0.998 | 1.000 |
| HepG2 | genotoxicity | Tail_moment | Encapsulated Bombex Farumy | 500.00 | 8.81 (5.584) | 0.606 | 1.000 |
| HepG2 | genotoxicity | Tail_moment | Encapsulated Bombex Farumy | 1,000.00 | 16.64 (5.584) | 0.039 | 0.118 |
| HepG2 | genotoxicity | Tail_moment | Released Bombex Farumy | 0.01 | 5.79 (9.428) | 0.997 | 1.000 |
| HepG2 | genotoxicity | Tail_moment | Released Bombex Farumy | 0.10 | 11.57 (7.921) | 0.694 | 1.000 |
| HepG2 | genotoxicity | Tail_moment | Released Bombex Farumy | 1.00 | 5.78 (7.921) | 0.990 | 1.000 |
| HepG2 | genotoxicity | Tail_moment | Released Bombex Farumy | 10.00 | 16.89 (7.921) | 0.262 | 0.940 |
| HepG2 | genotoxicity | Tail_moment | Released Bombex Farumy | 100.00 | 6.74 (7.048) | 0.953 | 1.000 |
| HepG2 | genotoxicity | Tail_moment | Released Bombex Farumy | 500.00 | 10.10 (7.048) | 0.714 | 1.000 |
| HepG2 | genotoxicity | Tail_moment | Released Bombex Farumy | 1,000.00 | 31.68 (7.048) | <0.001 | 0.002 |
| HL60 | cytotoxicity | % Viability | Positive Control (100 μM H2O2) | 100.00 | -47.20 (10.043) | 0.039 | n/a |
| HepG2 | cytotoxicity | % Viability | Positive Control (100 μM H2O2) | 100.00 | -10.10 (2.709) | 0.091 | n/a |
| HL60 | genotoxicity | DNA_in_Ta | Positive Control (100 μM H2O2) | 100.00 | 14.00 (4.819) | 0.048 | 0.048 |
| HL60 | genotoxicity | Tail_Length | Positive Control (100 μM H2O2) | 100.00 | 32.30 (4.596) | 0.015 | 0.046 |
| HL60 | genotoxicity | Tail_moment | Positive Control (100 μM H2O2) | 100.00 | 12.03 (1.702) | 0.004 | 0.017 |
| HL60 | genotoxicity | Olive_moment | Positive Control (100 μM H2O2) | 100.00 | 4.34 (1.105) | 0.020 | 0.046 |
| HepG2 | genotoxicity | DNA_in_Ta | Positive Control (100 μM H2O2) | 100.00 | 45.70 (3.492) | 0.005 | 0.014 |
| HepG2 | genotoxicity | Tail_Length | Positive Control (100 μM H2O2) | 100.00 | 57.40 (5.083) | <0.001 | 0.002 |
| HepG2 | genotoxicity | Tail_moment | Positive Control (100 μM H2O2) | 100.00 | 57.92 (5.566) | 0.038 | 0.064 |
| HepG2 | genotoxicity | Olive_moment | Positive Control (100 μM H2O2) | 100.00 | 22.19 (1.793) | 0.032 | 0.064 |
